# Supplementary figures and images for: Application of non-invasive low-intensity pulsed electric field with thermal cycling-hyperthermia for synergistically enhanced anticancer effect of chlorogenic acid on PANC-1 cells
Source: PLoS One. 2020 Jan 29;15(1):e0222126. doi: 10.1371/journal.pone.0222126 (PMC6988950; doi:10.1371/journal.pone.0222126)

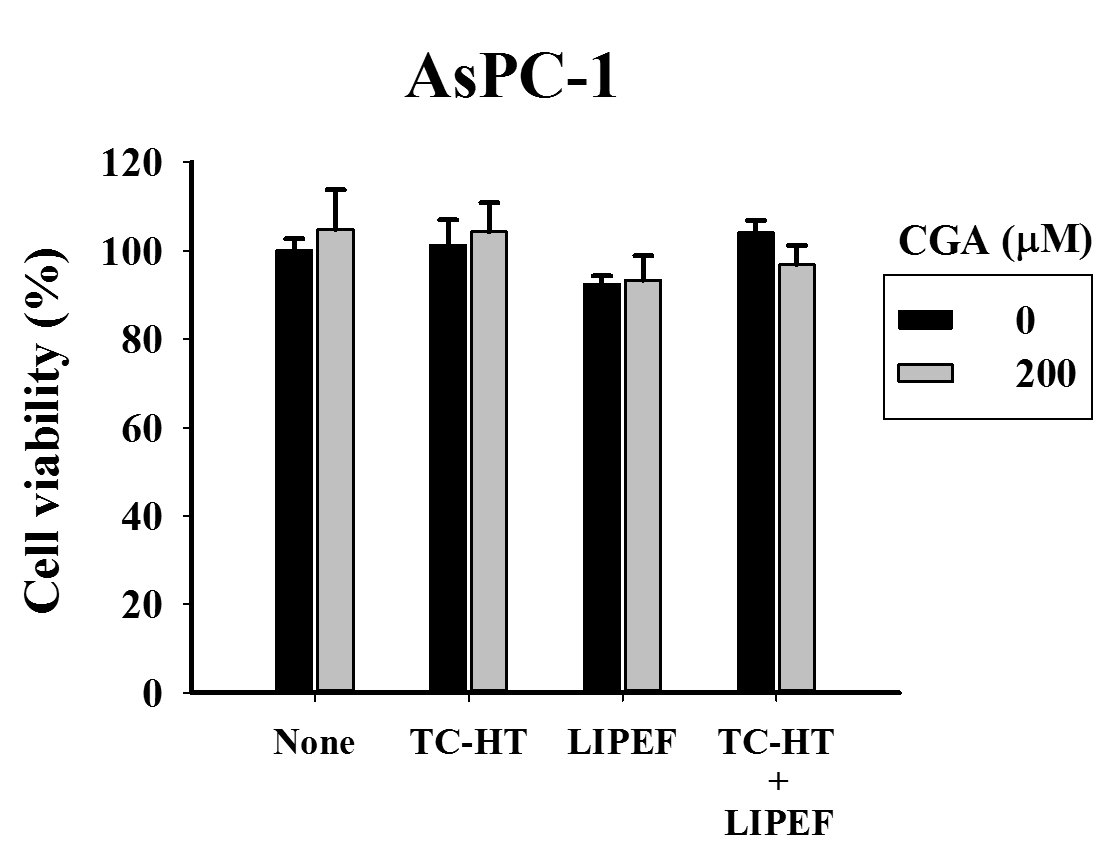

Supplement: S1 Fig — Data are presented as mean ± S.D. in triplicate. (TIFF) [file pone.0222126.s001.tiff]

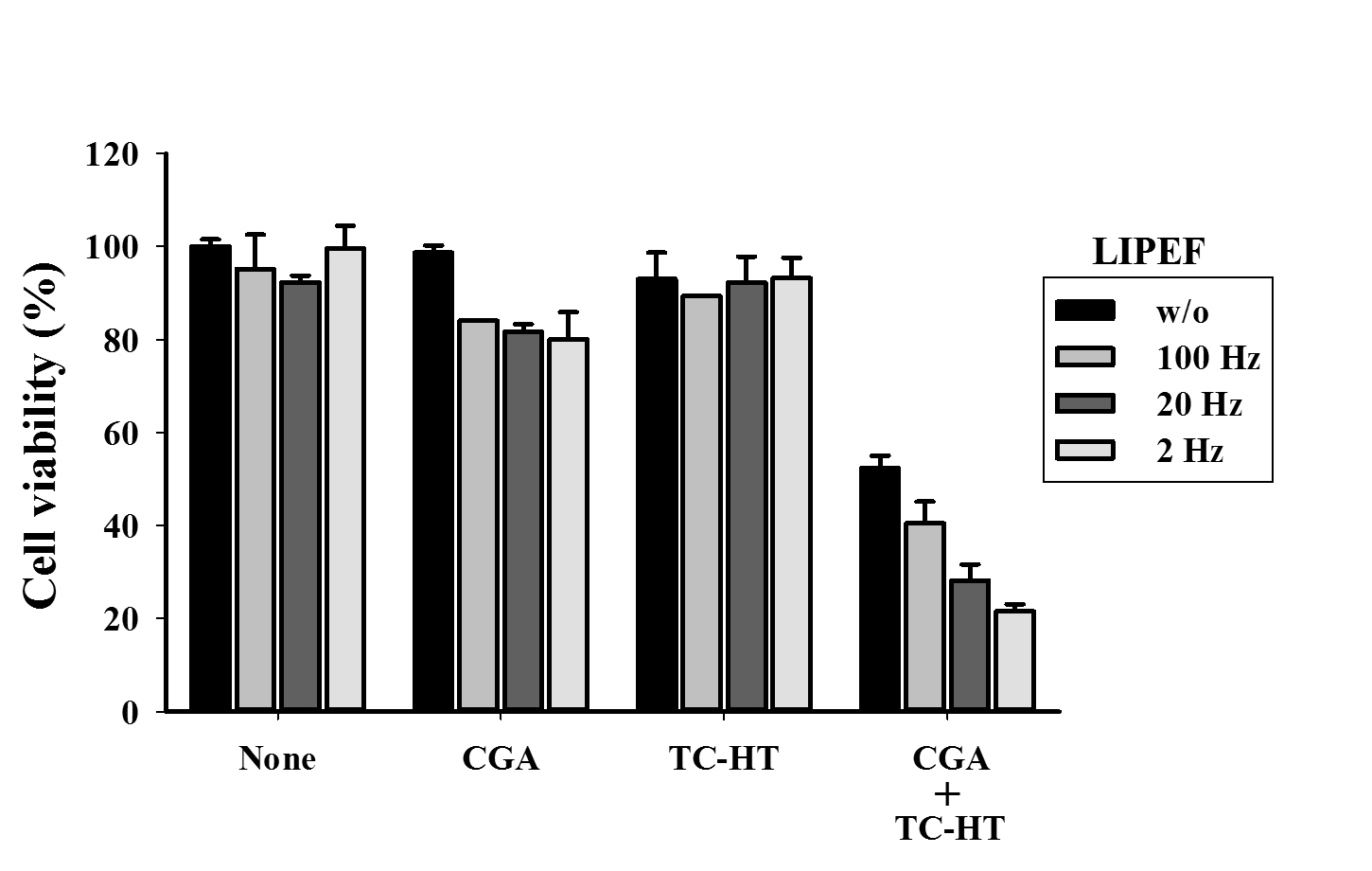

Supplement: S2 Fig — Data are presented as mean ± S.D. in triplicate. (TIFF) [file pone.0222126.s002.tiff]

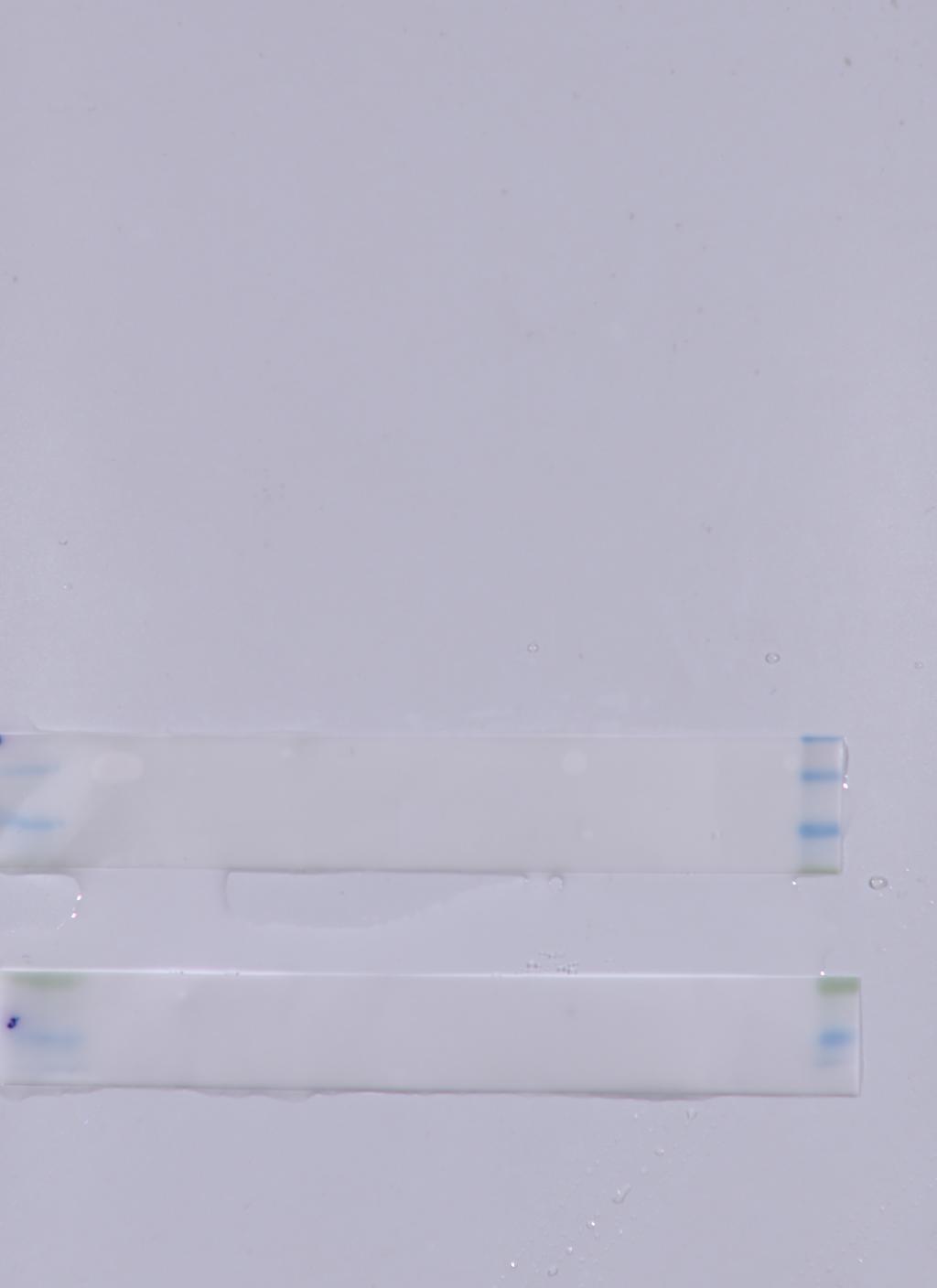

Supplement: S1 File — (ZIP) [file pone.0222126.s003.zip › original blots/lu-p53 p21 nac dr-db 2019.04.20_17.15.07_Ch-Marker.jpg]

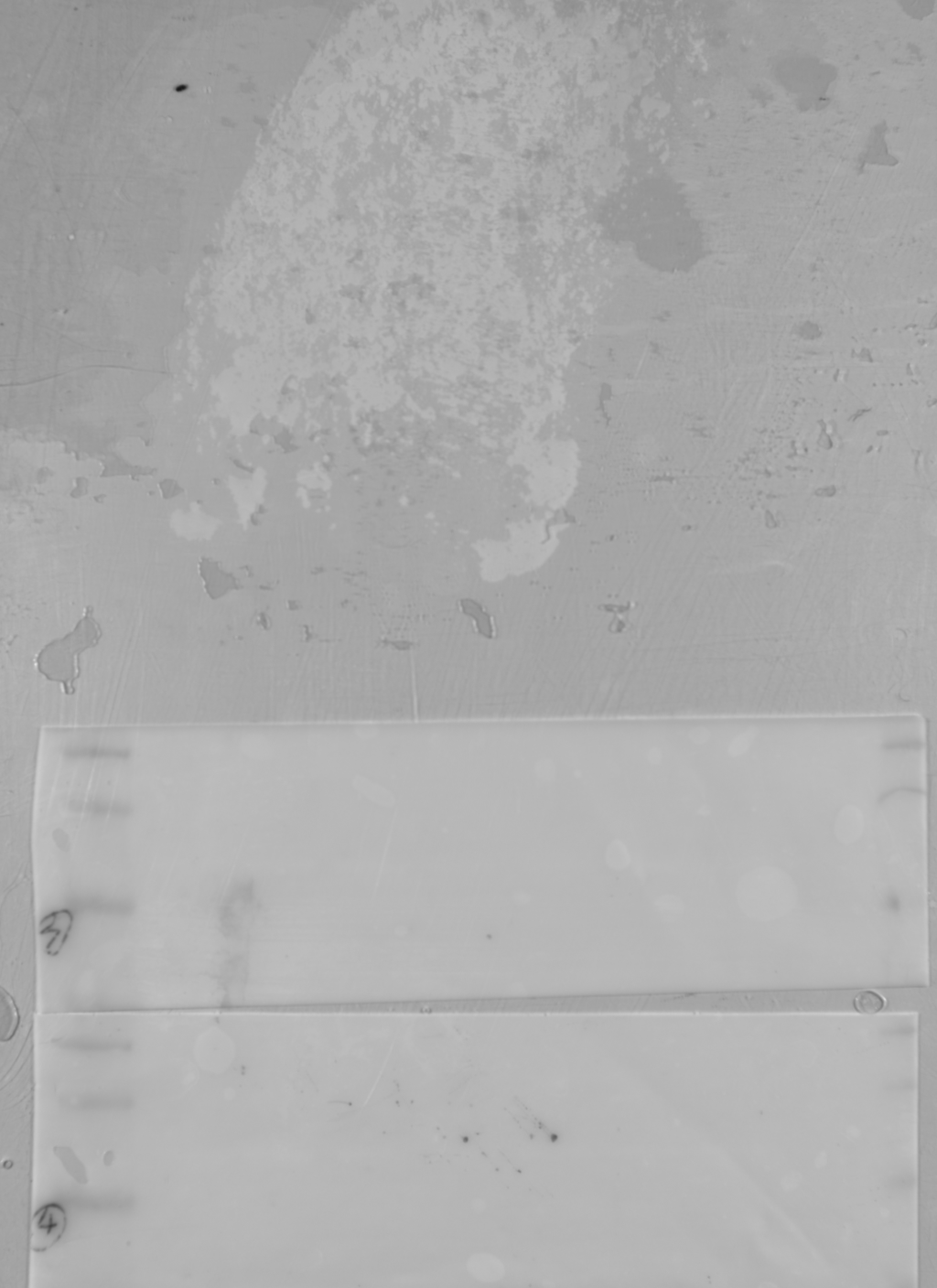

Supplement: S1 File — (ZIP) [file pone.0222126.s003.zip › original blots/lu-GAPDH nac 2019.03.21_18.06.31_Ch-Marker.tif]

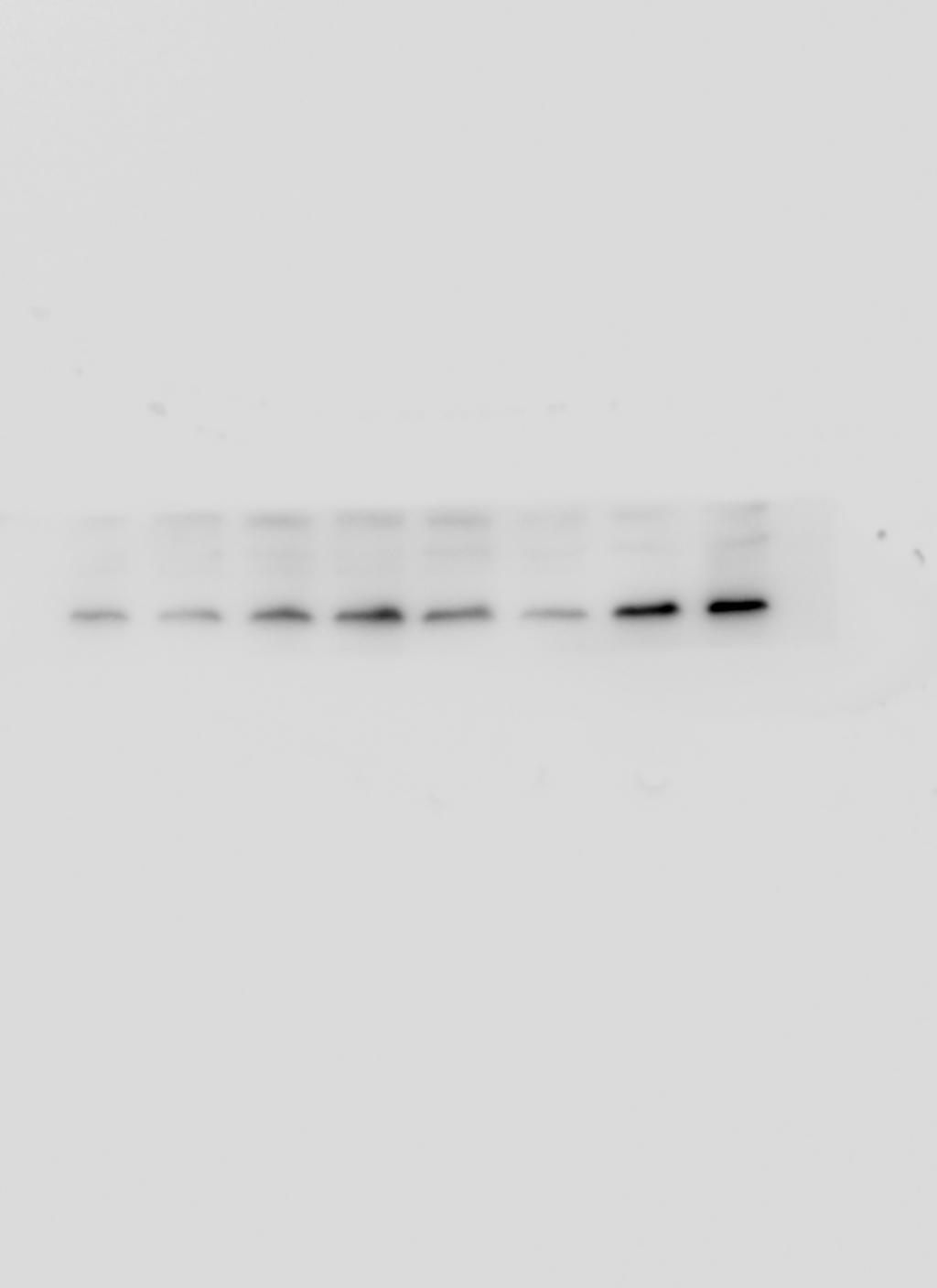

Supplement: S1 File — (ZIP) [file pone.0222126.s003.zip › original blots/lu-p53 c1 2019.05.08_16.46.38-01_Ch.jpg]

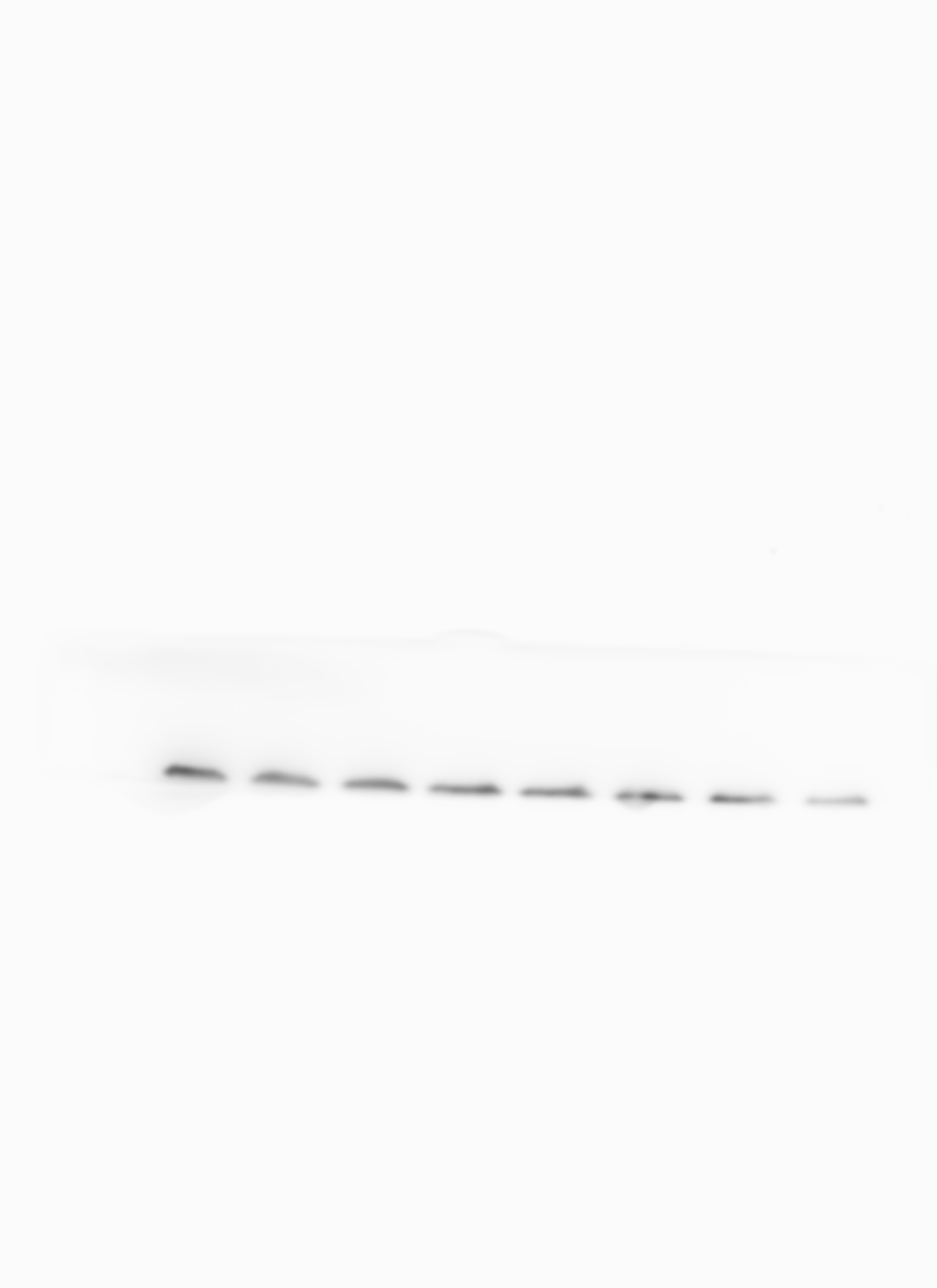

Supplement: S1 File — (ZIP) [file pone.0222126.s003.zip › original blots/lu-bcl2 c1 2019.05.10_15.13.05_Ch.tif]

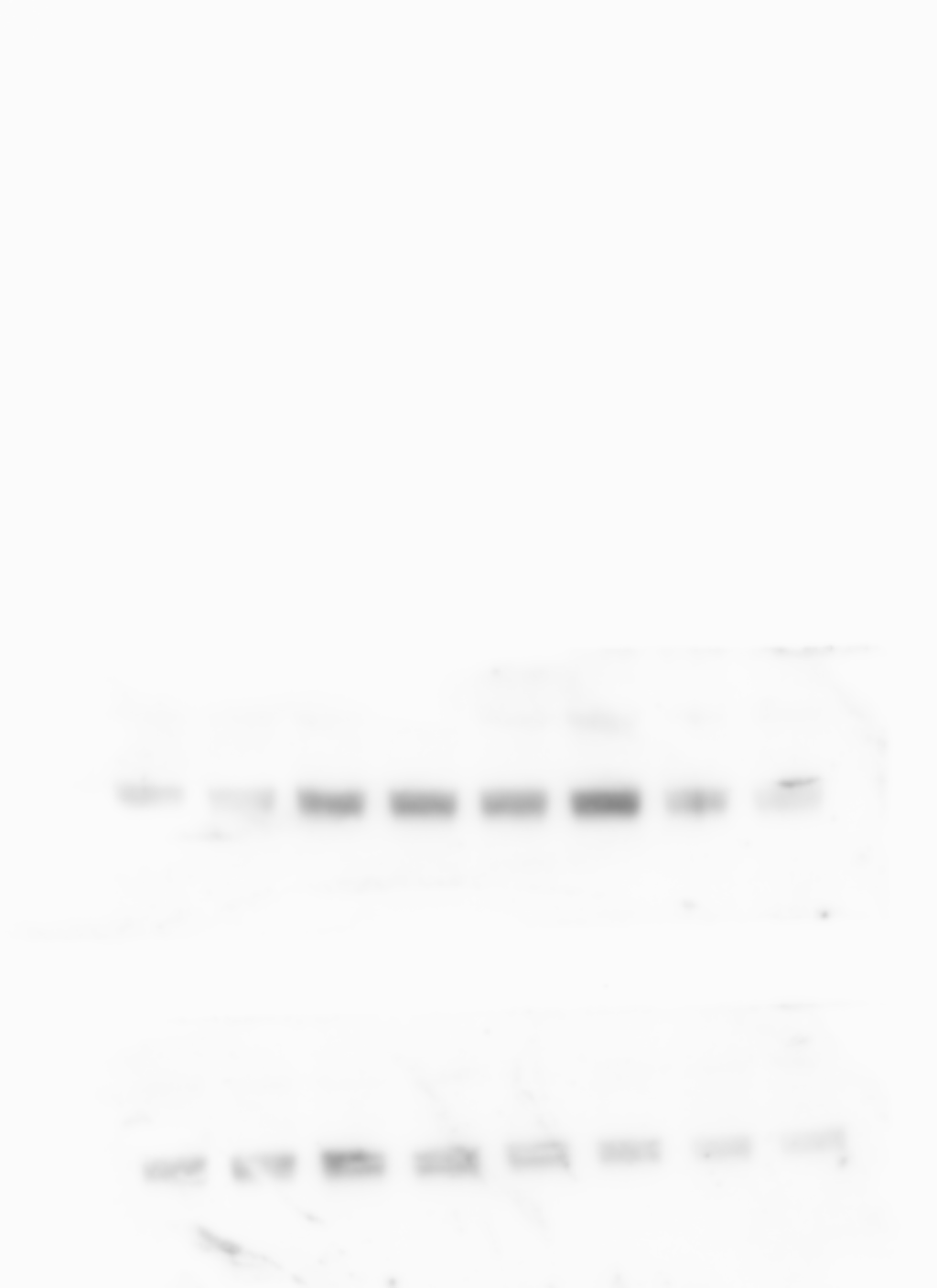

Supplement: S1 File — (ZIP) [file pone.0222126.s003.zip › original blots/lu-p53 nac 2019.03.19_15.31.09_Ch.tif]

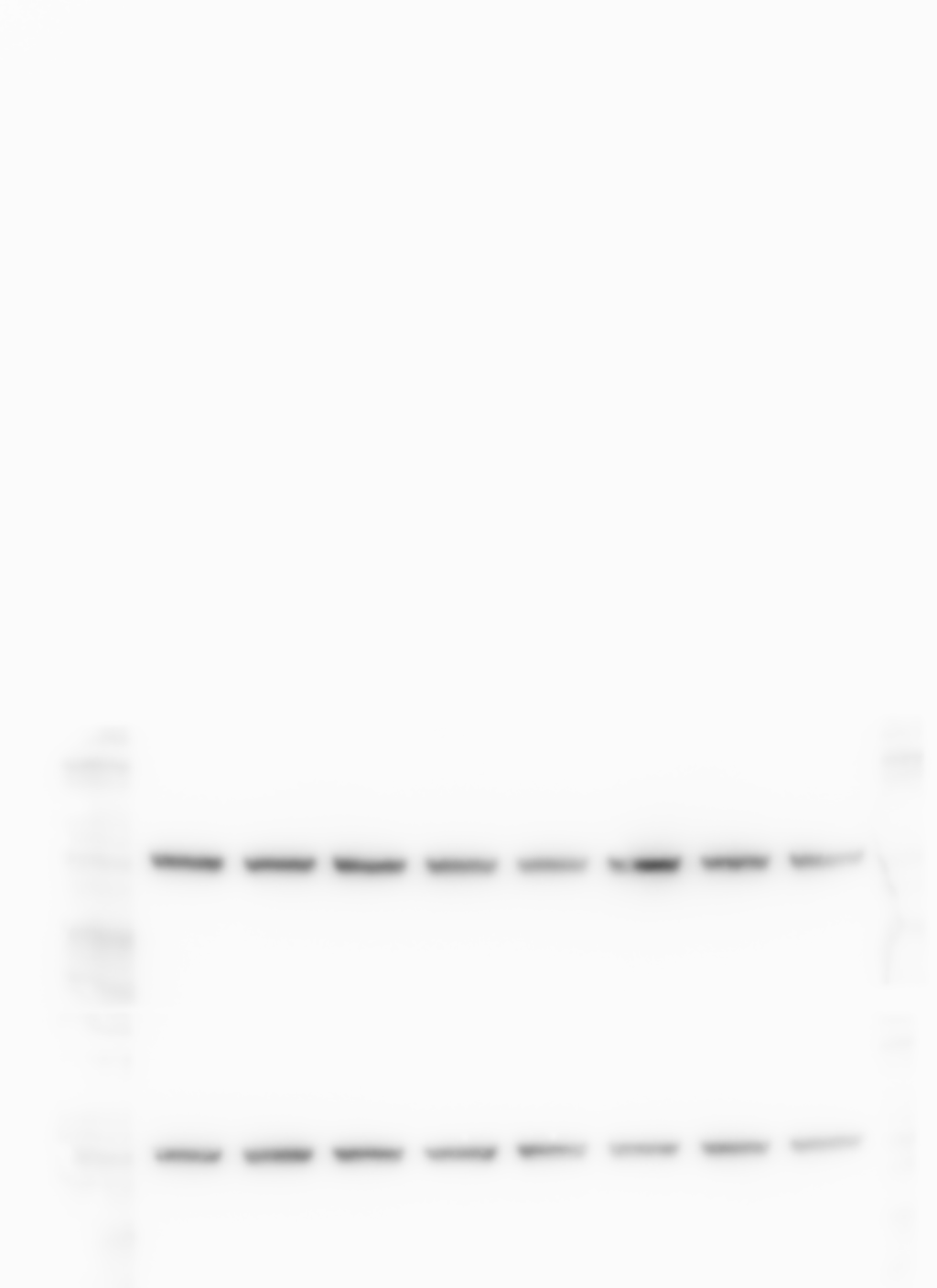

Supplement: S1 File — (ZIP) [file pone.0222126.s003.zip › original blots/lu-GAPDH nac 2019.03.21_18.06.31_Ch.tif]

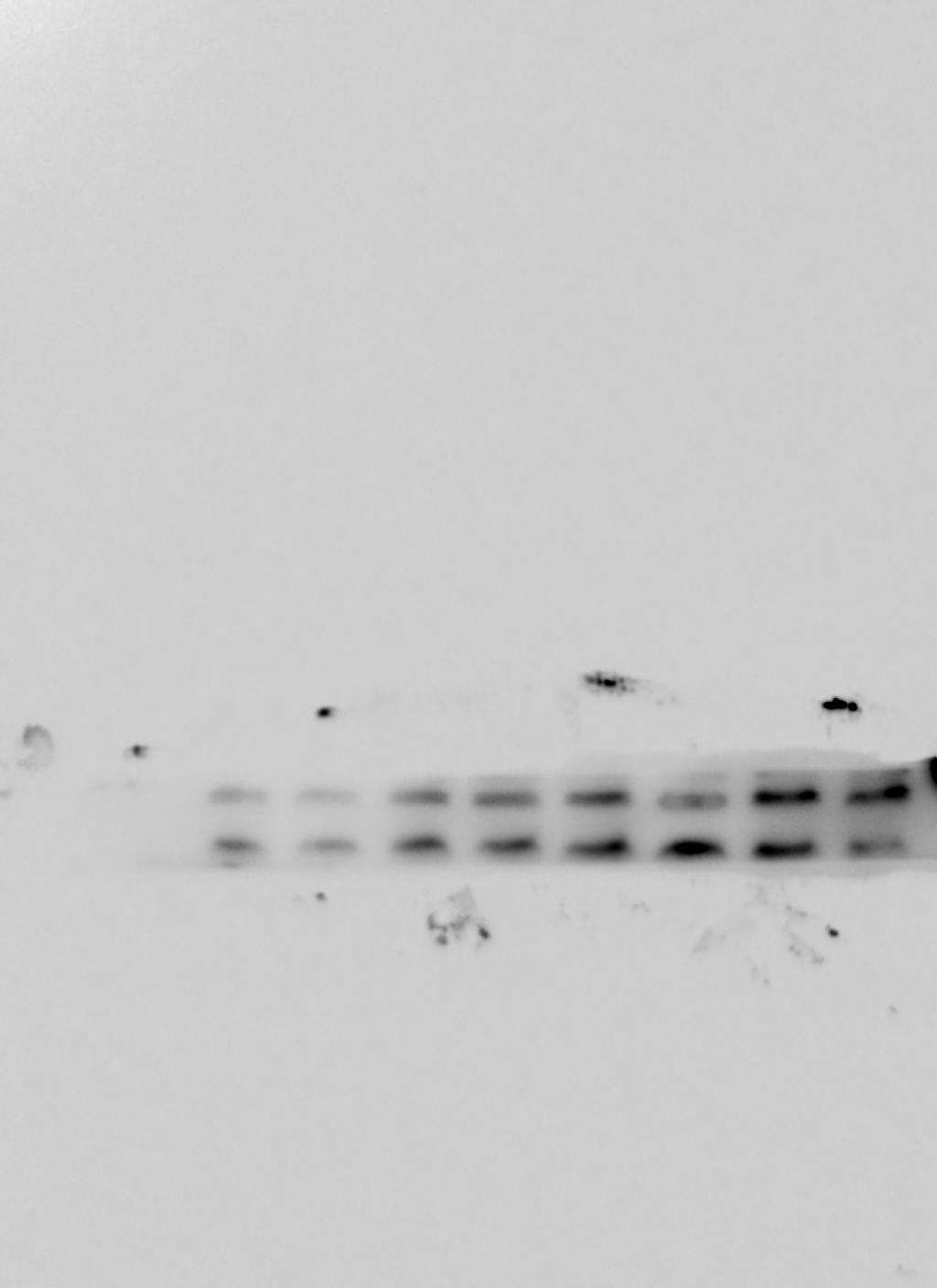

Supplement: S1 File — (ZIP) [file pone.0222126.s003.zip › original blots/lu-p21 c1 2019.05.08_16.52.27-03_Ch.jpg]

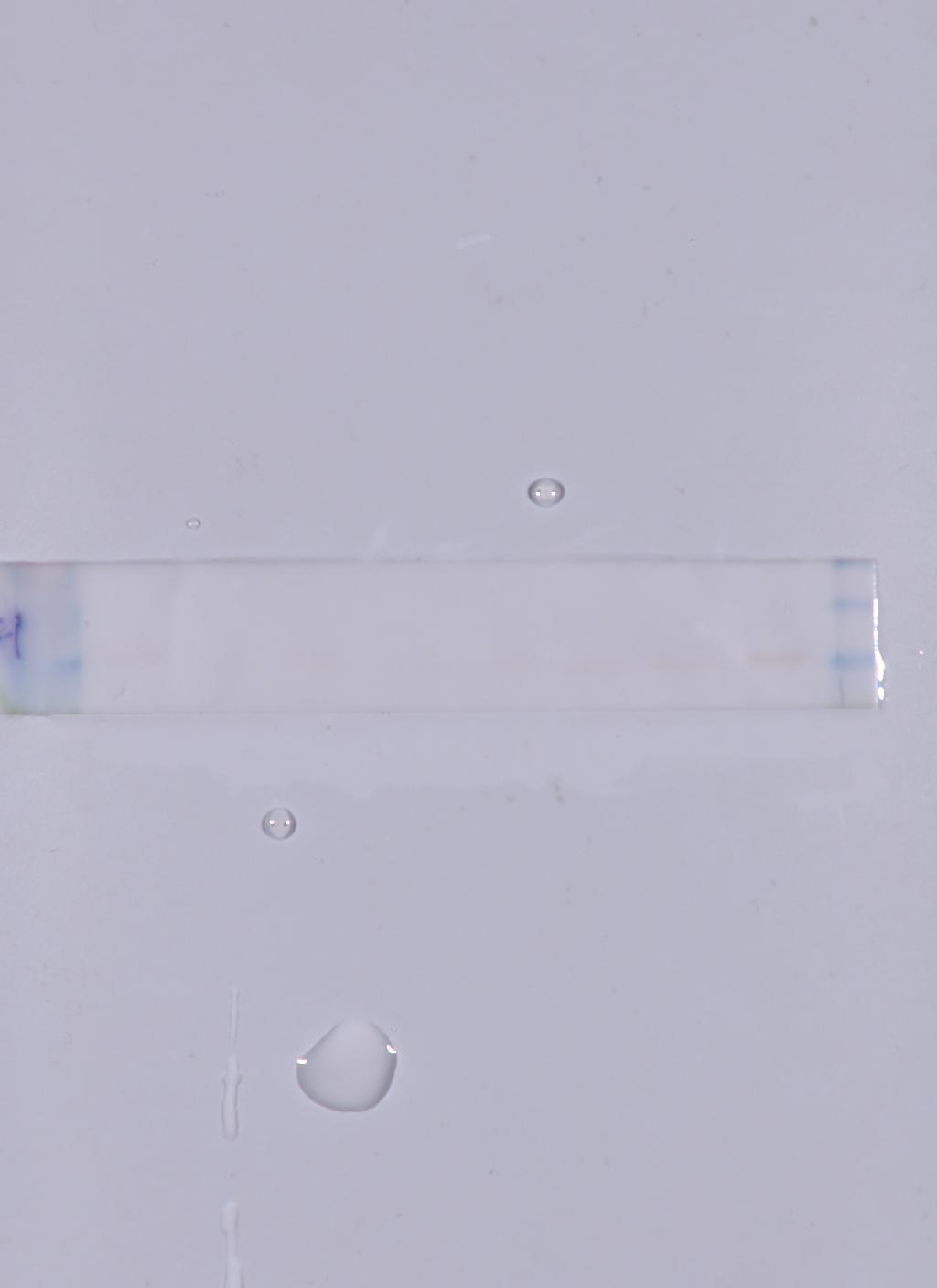

Supplement: S1 File — (ZIP) [file pone.0222126.s003.zip › original blots/lu-GAPDH C1 2019.05.15_16.55.53_Ch-Marker.jpg]

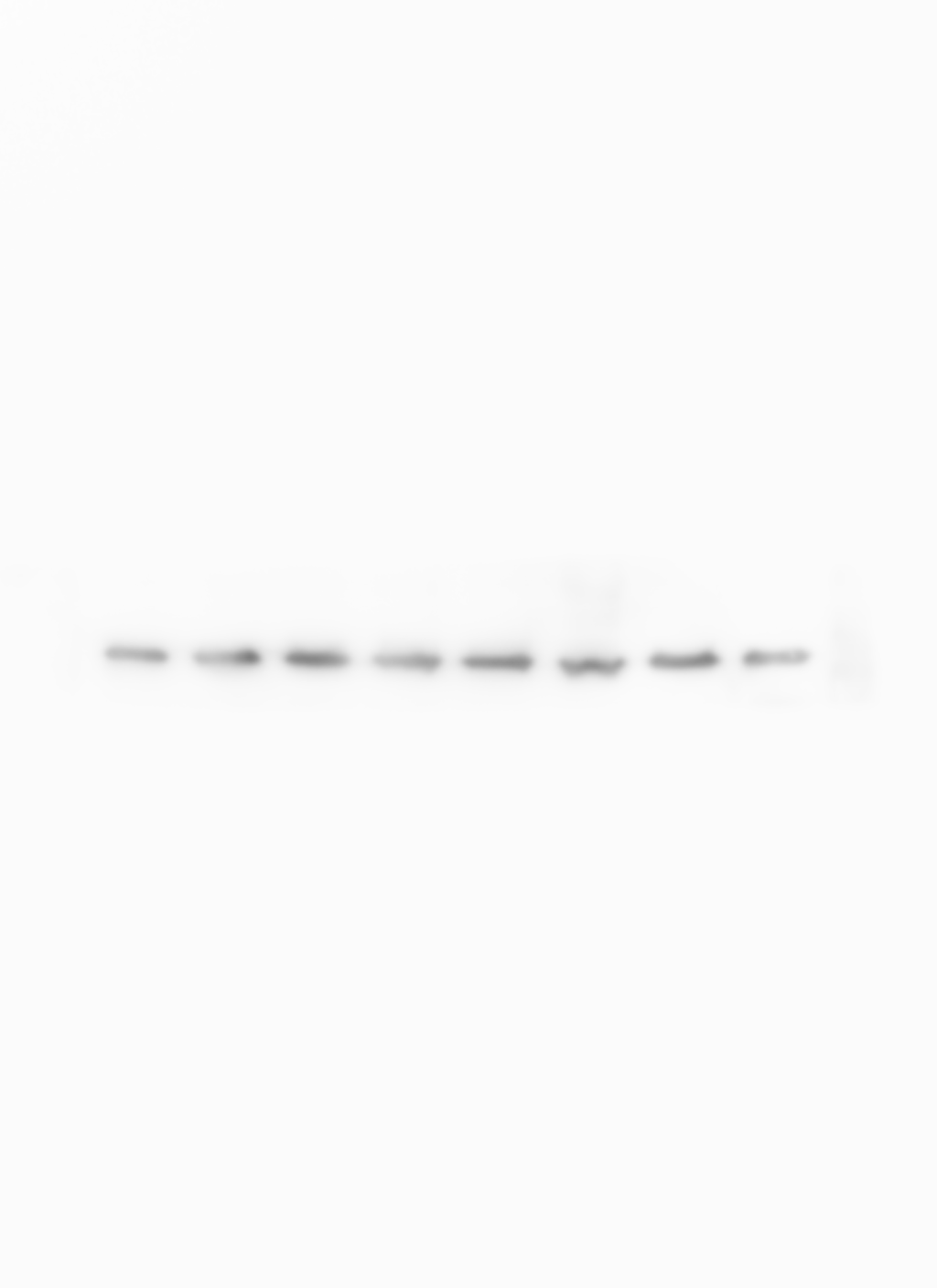

Supplement: S1 File — (ZIP) [file pone.0222126.s003.zip › original blots/lu-GAPDH C1 2019.05.15_16.55.53_Ch.tif]

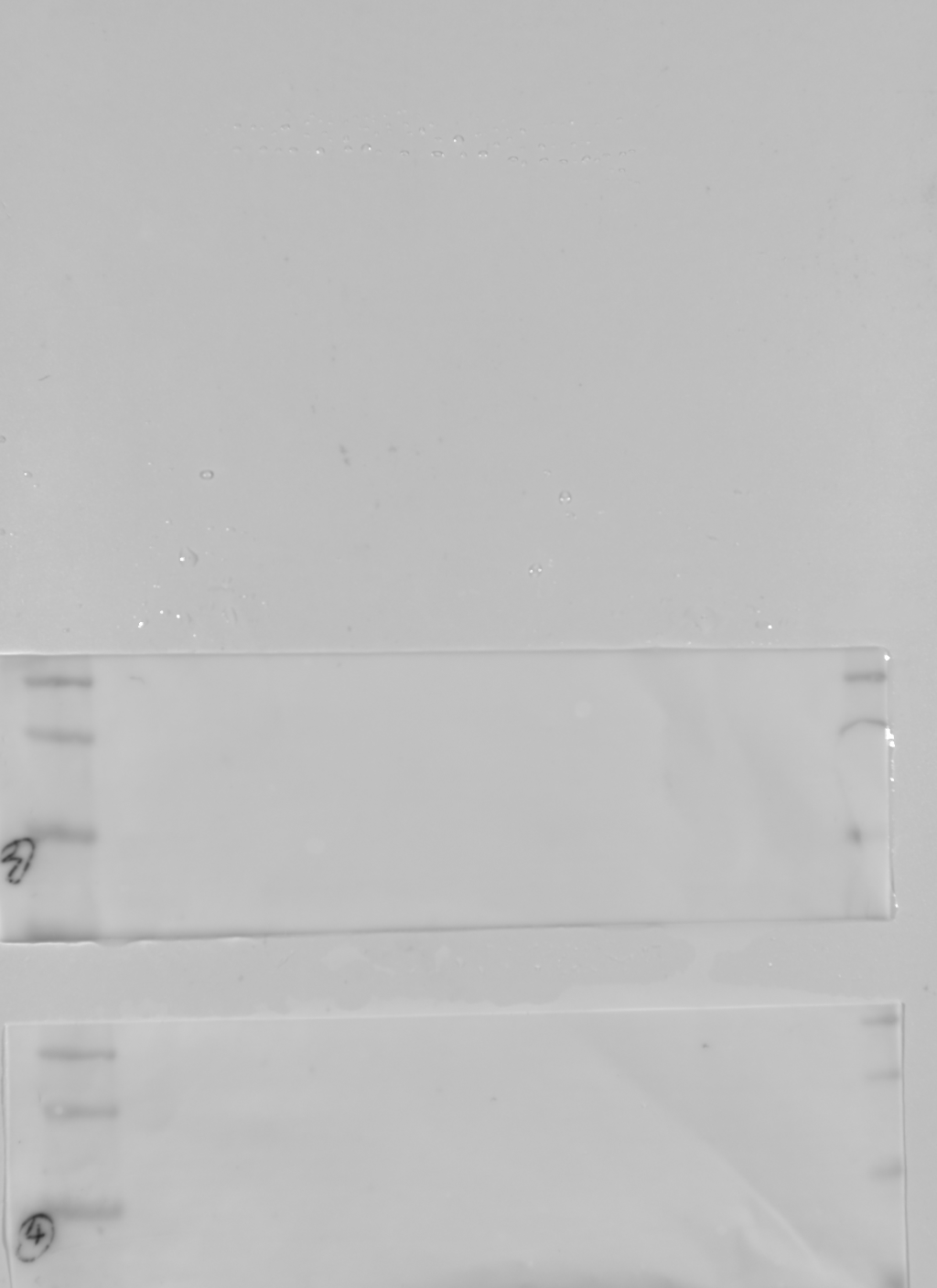

Supplement: S1 File — (ZIP) [file pone.0222126.s003.zip › original blots/lu-p53 nac 2019.03.19_15.31.09_Ch-Marker.tif]

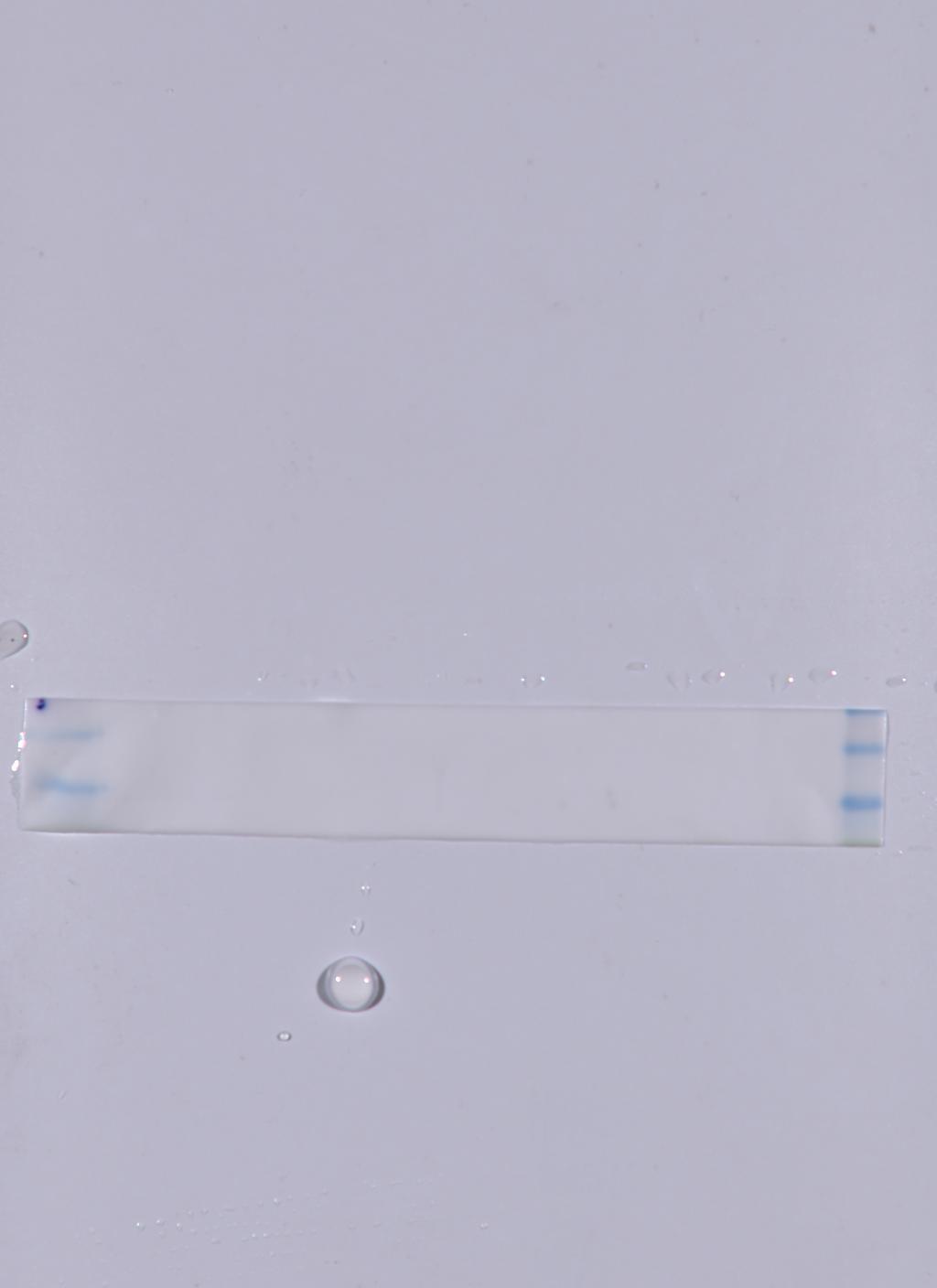

Supplement: S1 File — (ZIP) [file pone.0222126.s003.zip › original blots/lu-Bcl-2 nac dr db 2019.04.22_14.54.56_Ch-Marker.jpg]

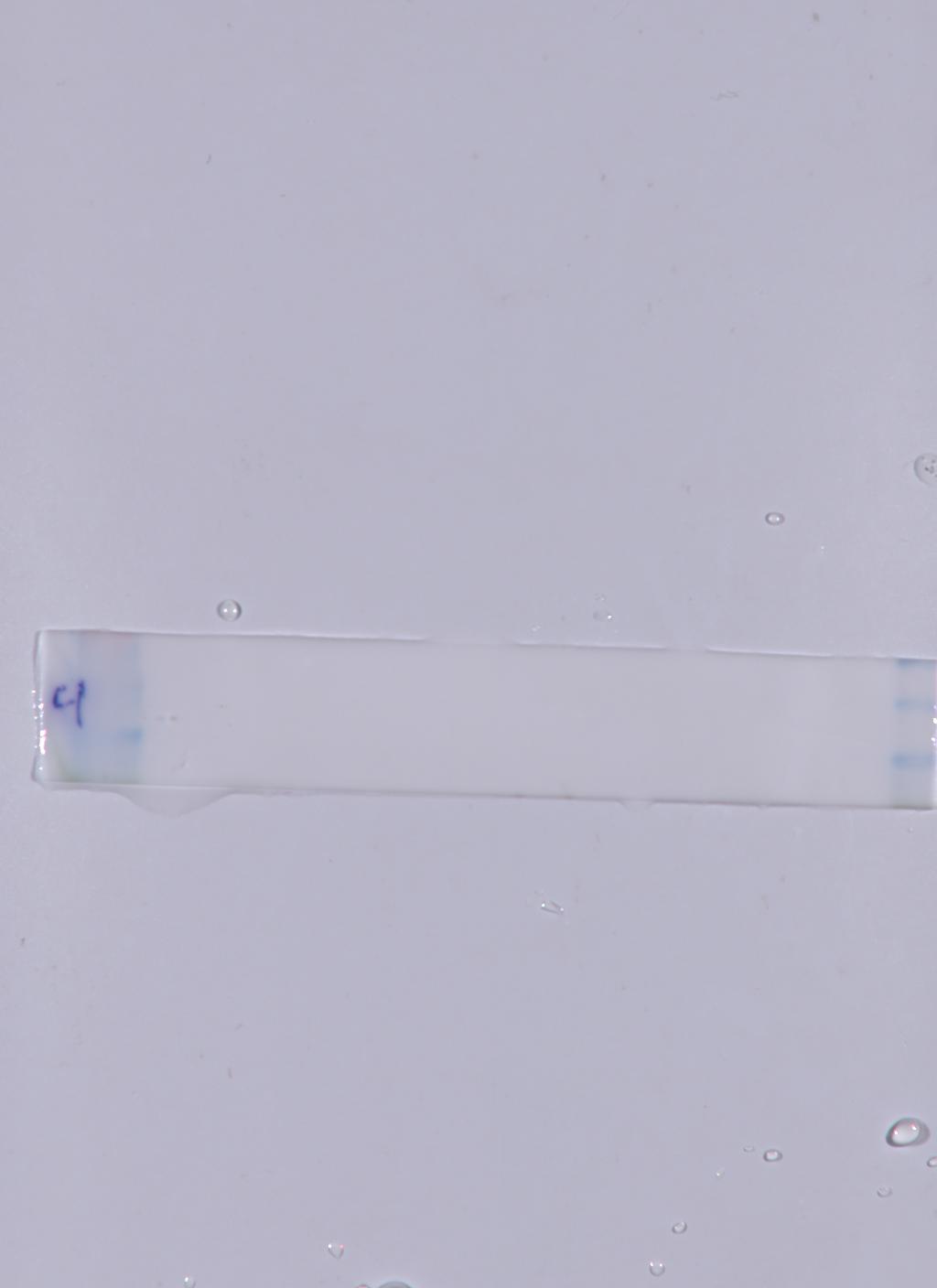

Supplement: S1 File — (ZIP) [file pone.0222126.s003.zip › original blots/lu-bcl2 c1 2019.05.10_15.13.05_Ch-Marker.jpg]

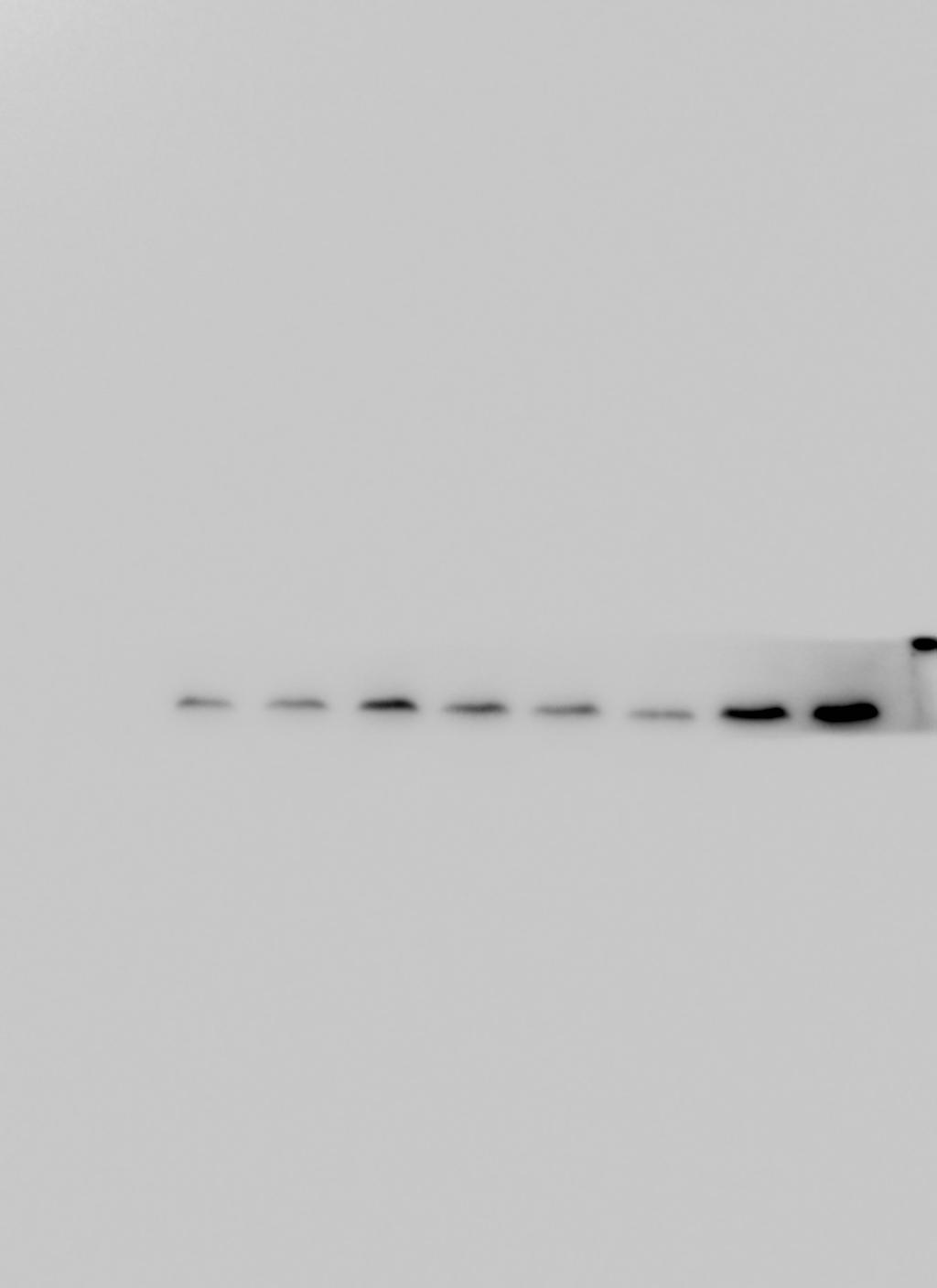

Supplement: S1 File — (ZIP) [file pone.0222126.s003.zip › original blots/lu-bax c1 2019.05.13_17.03.04-01_Ch.jpg]

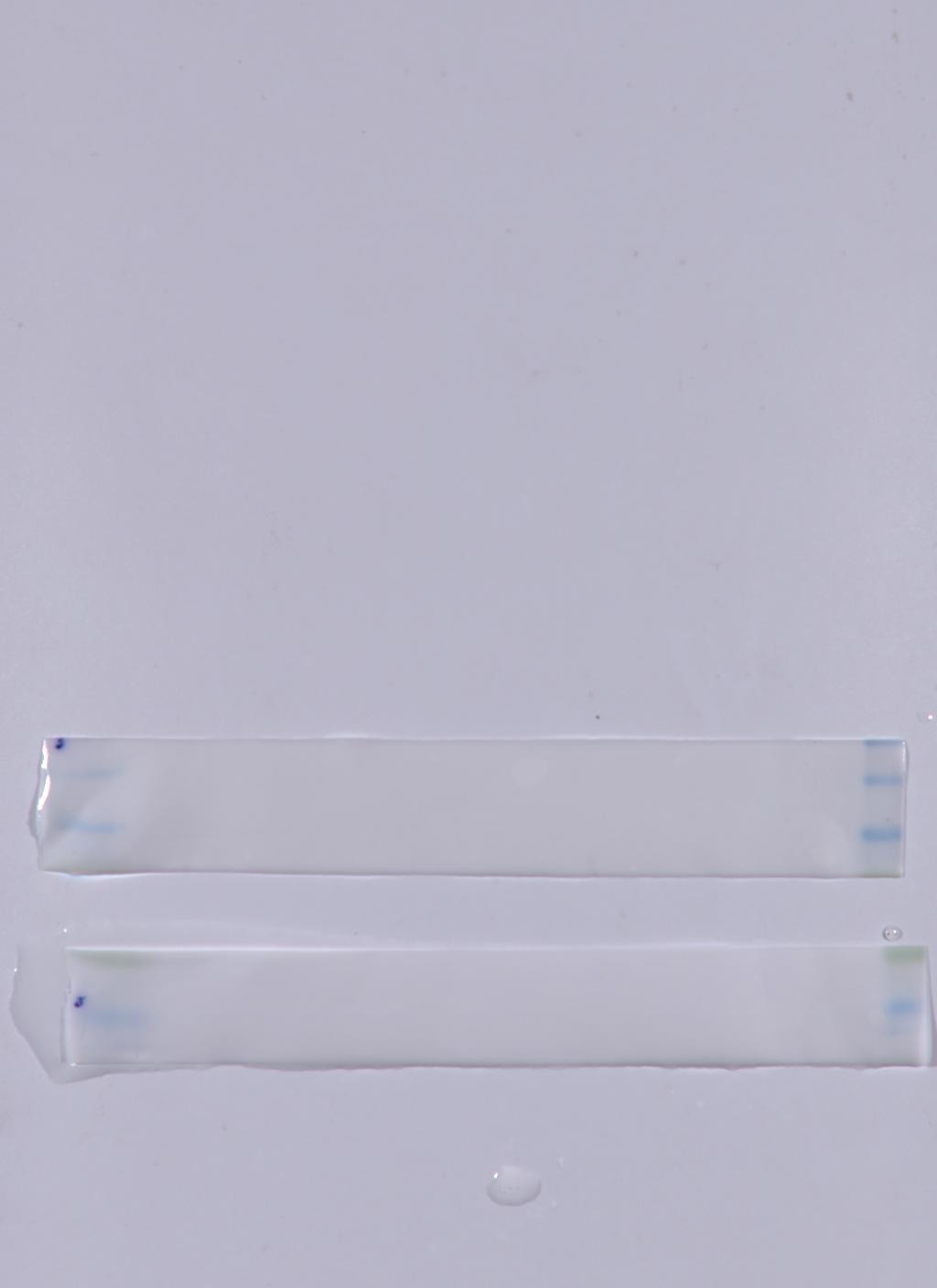

Supplement: S1 File — (ZIP) [file pone.0222126.s003.zip › original blots/lu-Bax nac dr db 2019.04.22_14.51.01_Ch-Marker.jpg]

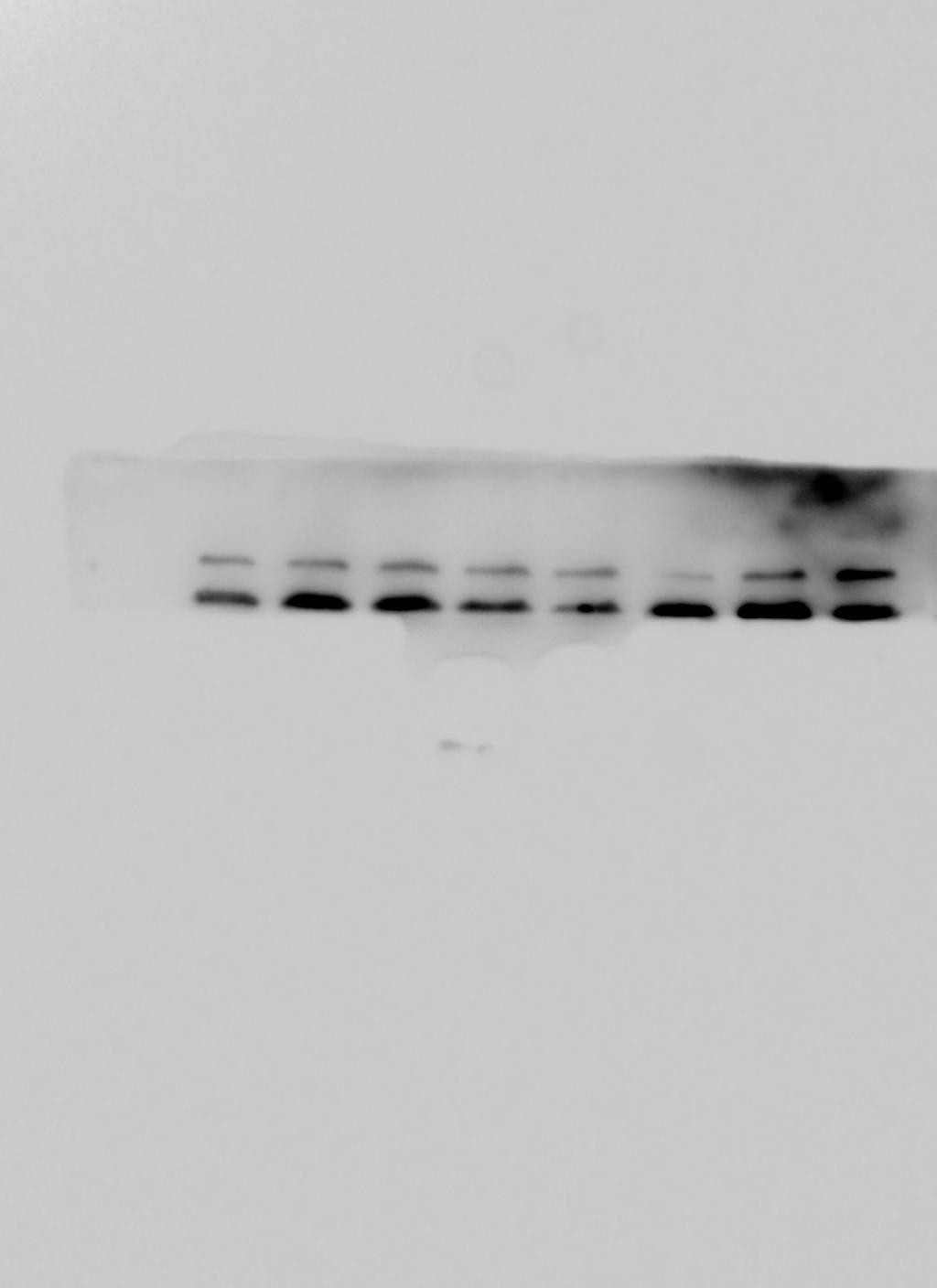

Supplement: S1 File — (ZIP) [file pone.0222126.s003.zip › original blots/lu-cas9 c1 2019.05.13_16.45.22-02_Ch.jpg]
