# Supplementary material for: Application of non-invasive low-intensity pulsed electric field with thermal cycling-hyperthermia for synergistically enhanced anticancer effect of chlorogenic acid on PANC-1 cells
Source: PLoS One. 2020 Jan 29;15(1):e0222126. doi: 10.1371/journal.pone.0222126 (PMC6988950; doi:10.1371/journal.pone.0222126)
Supplement: S2 File — (PDF) [file pone.0222126.s004.pdf]

		C			100			200			300	
	0.5837	0.594	0.5854	0.5792	0.5711	0.574	0.5433	0.581	0.5367	0.5049	0.5549	0.5495
	0.5886	0.5851	0.6171	0.5848	0.5928	0.5822	0.554	0.5995	0.5204	0.551	0.5664	0.5199
	0.58615	0.58955	0.60125	0.582	0.58195	0.5781	0.54865	0.59025	0.52855	0.52795	0.56065	0.5347
Average	0.592317			0.580683			0.555817			0.5411	0.017264	
SD	0.007921			0.002237			0.029416					
		PEF			100P			200P			300P	
	0.5719	0.56	0.5568	0.529	0.5479	0.5156	0.4922	0.5273	0.51	0.4261	0.4643	0.4844
	0.561	0.5445	0.5582	0.5424	0.5508	0.5166	0.4917	0.517	0.528	0.46	0.4643	0.485
	0.56645	0.55225	0.5575	0.5357	0.54935	0.5161	0.49195	0.52215	0.519	0.44305	0.4643	0.4847
Average	0.558733			0.533717			0.511033			0.464017		
SD	0.00718			0.016713			0.016602			0.020826		
		TC			100TC			200TC			300TC	
	0.5957	0.5799	0.5237	0.4854	0.4561	0.4912	0.3547	0.3297	0.3312	0.274	0.3173	0.2607
	0.6044	0.565	0.5447	0.5059	0.4635	0.5417	0.3532	0.3177	0.3224	0.2831	0.3285	0.2605
	0.60005	0.57245	0.5342	0.49565	0.4598	0.51645	0.35395	0.3237	0.3268	0.27855	0.3229	0.2606
Average	0.571383			0.490633			0.334817			0.28735		
SD	0.014248			0.028656			0.016642			0.032069		
		TC+PEF			100+TC+PEF			200+TC+PEF			300+TC+PEF	
	0.5657	0.5498	0.5403	0.4946	0.4944	0.4652	0.1145	0.1101	0.1154	0.1083	0.099	0.0959
	0.5622	0.5703	0.5204	0.3936	0.4721	0.4656	0.1204	0.117	0.1144	0.1032	0.1059	0.0877
	0.56395	0.56005	0.53035	0.4441	0.48325	0.4654	0.11745	0.11355	0.1149	0.10575	0.10245	0.0918
Average	0.54755			0.46425			0.117267			0.1		
SD	0.023537			0.0196			0.001981			0.007291		

	C			200	
0.095	0.093	0.093	0.099	0.103	0.107
0.093667	0.001155		0.103	0.004	
100	1.232776		109.9644	4.270463	

	PEF			200P	
0.096	0.09	0.088	0.098	0.102	0.1
0.091333	0.004163		0.1	0.002	
97.5089	4.444838		106.7616	2.135231	

	TC			200TC	
0.0896	0.0924	0.094	0.0915	0.0918	0.0939
0.092	0.002227		0.0924	0.001308	
98.22064	2.377693		98.64769	1.396089	

	TC+PEF			200+TC+PEF	
0.0944	0.099	0.099	0.09	0.102	0.103
0.097467	0.002656		0.098333	0.007234	
104.0569	2.835386		104.9822	7.723322	

								+NAC			
	C			200			C			200	
0.5699	0.5899	0.55425	0.5785	0.5119	0.5942	0.55655	0.5676	0.53215	0.5793	0.6248	0.58675
0.57135	0.017869		0.561533	0.043695		0.5521	0.018139		0.59695	0.024405	
100	3.127536		98.28185	7.647617		96.63079	3.174782		104.4806	4.271421	
	PEF			200P			PEF			200P	
0.58	0.5942	0.6019	0.4951	0.495	0.4747	0.5919	0.59095	0.62415	0.5457	0.5668	0.5518
0.592033	0.01111		0.488267	0.011749		0.602333	0.0189		0.554767	0.010858	
103.6201	1.944448		85.45842	2.05639		105.4228	3.307912		97.09752	1.900469	
	TC			200TC			TC			200TC	
0.55495	0.54255	0.5385	0.3267	0.2978	0.25475	0.53535	0.5488	0.59195	0.5916	0.58315	0.54
0.545333	0.008571		0.293083	0.036206		0.5587	0.02957		0.571583	0.027676	
95.44646	1.500119		51.29664	6.336949		97.78595	5.175499		100.0408	4.844029	
	TC+PEF			200+TC+PEF			TC+PEF			200+TC+PEF	
0.51375	0.56475	0.6389	0.204	0.14355	0.13055	0.58035	0.57035	0.56925	0.63305	0.6131	0.59755
0.572467	0.062931		0.159367	0.039196		0.573317	0.006116		0.614567	0.017795	
100.1954	11.01441		27.893	6.860298		100.3442	1.070417		107.564	3.114621	

				AVG	SD
C				100	0
P	88.18898	87.73585	107.5472	94.49067	11.30953
CGA	98.0315	86.61417	83.00654	89.2174	7.843466
200P	72.54902	89.37008	78.74016	80.21975	8.507579
H	93.27731	101.3072	90.15748	94.91399	5.752221
HP	88.23529	96.73203	86.22047	90.39593	5.578929
200H	37.73585	25.98425	29.13386	30.95132	6.082958
200HP	13.72549	2.362205	13.77953	9.955741	6.576251
