# Supplementary material for: Application of non-invasive low-intensity pulsed electric field with thermal cycling-hyperthermia for synergistically enhanced anticancer effect of chlorogenic acid on PANC-1 cells
Source: PLoS One. 2020 Jan 29;15(1):e0222126. doi: 10.1371/journal.pone.0222126 (PMC6988950; doi:10.1371/journal.pone.0222126)

C

G0/G1	S	G2/M
62.03	17.05	20.92
62.45	16.88	20.67
61.18	18.39	20.43
71.09	10.52	18.39
71.58	11.93	16.49

LIPEF

G0/G1	S	G2/M
61.43	14.13	24.45
60.41	15.81	23.78
58.69	22.45	18.86
57.46	19.57	22.97
55.18	21.01	23.81
66.82	17.99	15.19

TC

G0/G1	S	G2/M
59.26	15.44	25.31
46.95	28.95	24.1
57.21	24.77	18.02
56.19	20.19	23.62
60.56	16.22	23.23
57.03	15.94	27.03

TC+LIPEF

G0/G1	S	G2/M
47.78	28.22	24
44.63	25.84	29.52
50.85	11.46	37.69
51.15	20.66	28.19
58.2	16.2	25.6
55.34	18.95	25.71
57.13	21.31	21.56

CGA

G0/G1	S	G2/M
61.38	15.99	22.63
67.26	12.79	19.95
52.51	24.84	22.65
54.67	19.6	25.73
66.9	10.88	22.22
66.99	15.18	17.83

CGA+LIPEF

G0/G1	S	G2/M
60.34	13.98	25.68
53.87	23.67	22.46
53.56	20.84	25.6
51.92	21.22	26.86
56.2	19.02	24.77
61.93	17.83	20.24

CGA+TC

G0/G1	S	G2/M
42.67	20.39	36.95
46.69	17.96	35.35
37.21	20.87	41.92
47.15	15.59	37.26
52.28	17.03	30.68

CGA+TC+LIPEF

G0/G1	S	G2/M
42.61	16.28	41.1
39.59	14.62	45.79
46.21	15.9	37.89
39.78	17.23	43
45.5	17.74	36.76
49.11	17.82	33.07
52.43	14.2	33.36

Ctrl

	Annexin-/F	Annexin-/F	Annexin+/I	Annexin+/PI+	
	94.6	0.01	5.1	0.2	5.3
	94.1	0	5.4	0.4	5.8
	97	0.1	2.5	0.4	2.9
**Mean**	95.233	0.037	4.333	0.333	4.667
**SD**	1.550	0.055	1.595	0.115	1.550

TC

	Annexin-/F	Annexin-/F	Annexin+/I	Annexin+/PI+	
	91.4	0.1	8.1	0.4	8.5
	88.2	0.1	11	0.8	11.8
	87.4	0.1	11.6	0.9	12.5
**Mean**	89.000	0.100	10.233	0.900	10.933
**SD**	2.117	0.000	1.872	0.265	2.136

PEF

	Annexin-/F	Annexin-/F	Annexin+/I	Annexin+/PI+	
	91.7	0.1	7.6	0.6	8.2
	92.3	0.7	7.2	0.4	7.6
	94.5	0.8	4	0.8	4.8
**Mean**	92.833	0.533	6.267	0.600	6.867
**SD**	1.474	0.379	1.973	0.200	1.815

PEF+TC

	Annexin-/F	Annexin-/F	Annexin+/I	Annexin+/PI+	
	93.5	0.1	5.9	0.4	6.3
	83.2	0	16.4	0.5	16.9
	85.7	0.1	13.2	1	14.2
**Mean**	87.467	0.067	11.833	0.633	12.467
**SD**	5.372	0.058	5.382	0.321	5.508

CGA

	Annexin-/F	Annexin-/F	Annexin+/I	Annexin+/PI+	
	94.7	0.2	4.6	0.5	5.1
	96.6	0	2.6	0.5	3.1
	87.9	0.1	11.5	0.6	12.1
**Mean**	93.067	0.100	6.233	0.533	6.767
**SD**	4.574	0.100	4.669	0.058	4.726

CGA+TC

	Annexin-/F	Annexin-/F	Annexin+/I	Annexin+/PI+	
	58.1	0	39	2.8	41.8
	62	0.1	35.1	2.8	37.9
	52.8	1.4	32.8	14.6	47.4
**Mean**	57.633	0.500	35.633	6.733	42.367
**SD**	4.618	0.781	3.134	6.813	4.775

CGA+PEF

	Annexin-/F	Annexin-/F	Annexin+/I	Annexin+/PI+	
	87.6	0.1	11.6	0.7	12.3
	82.9	0	16.5	0.5	17
	74.8	0.1	22.8	2.3	25.1
**Mean**	81.767	0.067	16.967	1.167	18.133
**SD**	6.475	0.058	5.615	0.987	6.475

CGA+TC+PEF

	Annexin-/F	Annexin-/F	Annexin+/I	Annexin+/PI+	
	37.2	0.2	38.4	24.2	62.6
	33.6	0.2	38.1	28.1	66.2
	28.6	0.1	37.8	33.5	71.3
**Mean**	33.133	0.167	38.100	28.600	66.700
**SD**	4.319	0.058	0.300	4.670	4.371

	C	PEF	200	200PEF	TC-HT	TC-HT+PEF	200+TC-HT	200+TC-HT+PEF
	6.6	8.8	6.1	19.2	12.4	13.7	35.6	89.8
	7	9.5	8.1	21.8	12.7	14.2	39.7	85
	7.8	10.3	6.2	22.2	12.5	13.3	35.1	81.3
Mean	7.133333333	9.533333333	6.8	21.06666667	12.53333333	13.73333333	36.8	85.36666667
SD	0.611010093	0.75055535	1.126942767	1.628905563	0.152752523	0.450924975	2.523885893	4.261846235

ROS detection (DHE)

	C	P	200	200P	H	HP	200H	200HP
	1	0.8691589	0.7570093	1.6075949	1.271028	1.2803738	2.2803738	2.8130841
	1	1.2658228	1.1255725	1.6202532	1.1439492	1.2128618	2.2803738	2.7196262
	1	1.3544304	1.1485434	1.3831776	1.1623259	1.2082677	2.1495327	2.9906542
Mean	1	1.1631373	1.0103751	1.5370086	1.1924344	1.2338345	2.2367601	2.8411215
SD	0	0.2584189	0.2197216	0.1333718	0.0686815	0.0403697	0.0755412	0.1376721

FACSDiva Version 6.1.3

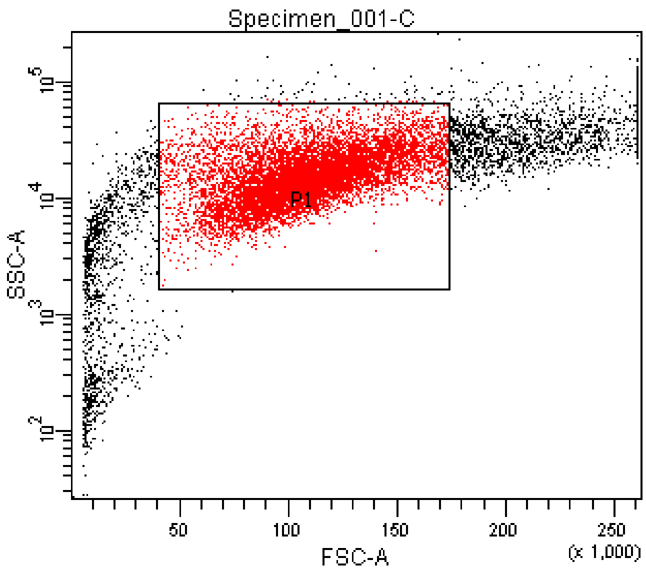

Experiment Name:	20180702 DHE TRI-3
Specimen Name:	Specimen_001
Tube Name:	C
Record Date:	Jul 2, 2018 3:02:22 PM
$OP:	Administrator
GUID:	5075bac9-8025-4f3a-bc34-fa...

Population	#Events	%Parent	PI-A Mean
P1	10,000	76.1	107

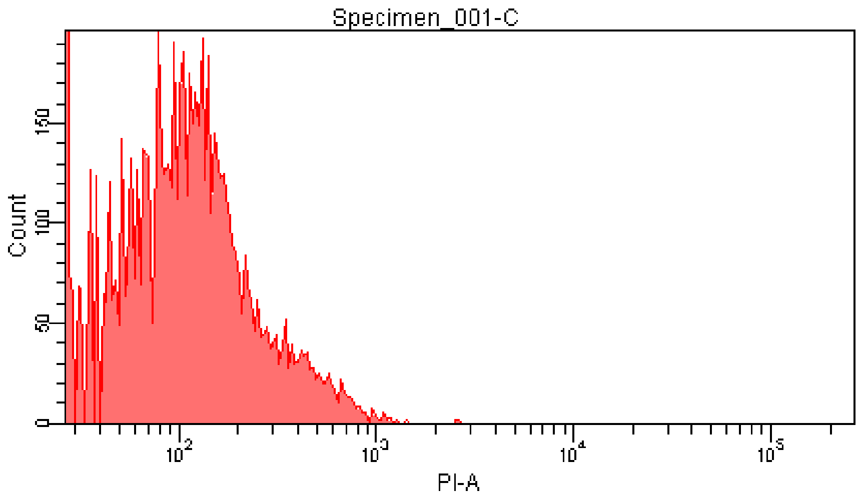

FACSDiva Version 6.1.3

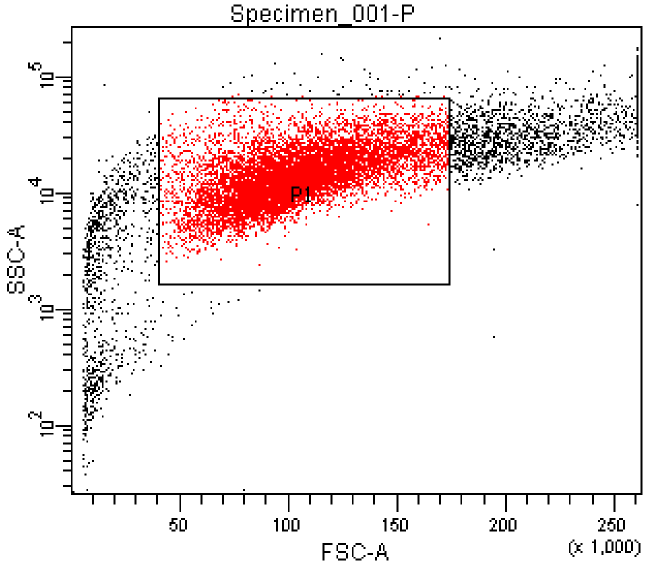

Experiment Name:	20180702 DHE TRI-3
Specimen Name:	Specimen_001
Tube Name:	P
Record Date:	Jul 2, 2018 3:01:14 PM
$OP:	Administrator
GUID:	4535a342-2bd1-4b66-911d-fe...

Population	#Events	%Parent	PI-A Mean
P1	10,000	79.2	93

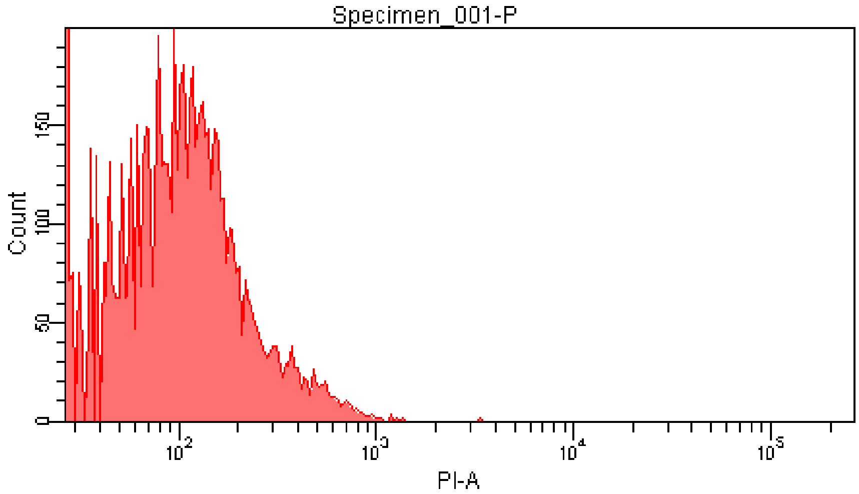

FACSDiva Version 6.1.3

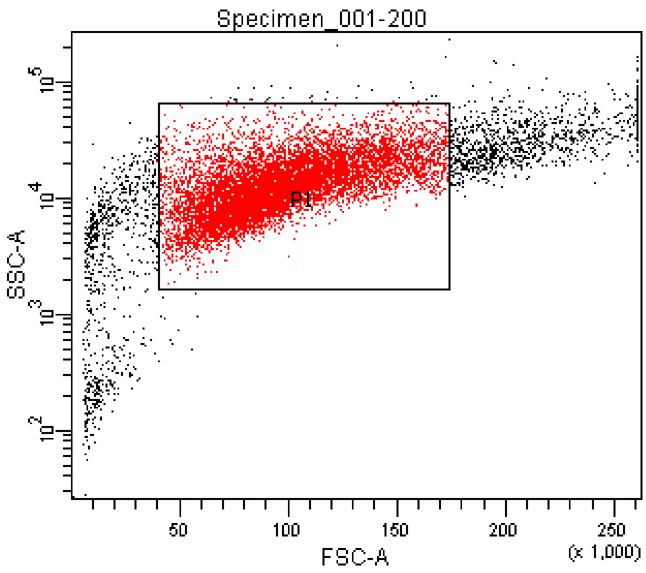

Experiment Name: 20180702 DHE TRI-3
Specimen Name: Specimen_001
Tube Name: 200
Record Date: Jul 2, 2018 3:00:15 PM
$OP: Administrator
GUID: a9f3f181-01f7-4563-b024-9d8...

Population	#Events	%Parent	PI-A Mean
P1	10,000	83.3	81

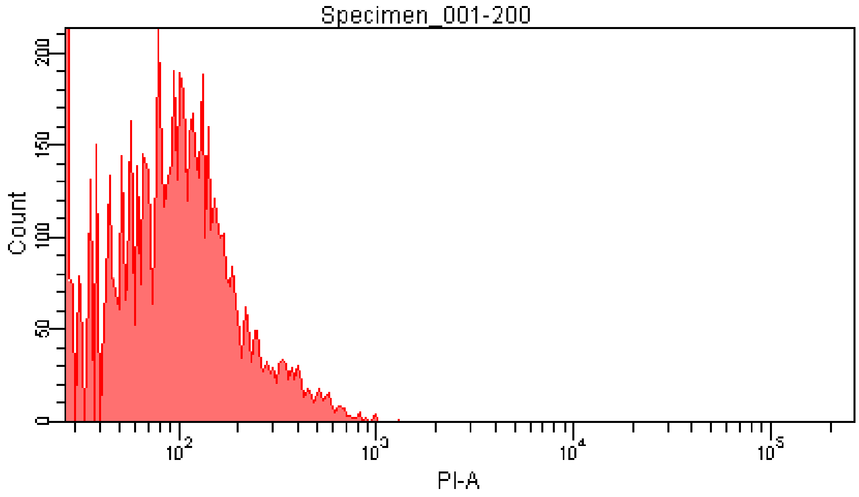

FACSDiva Version 6.1.3

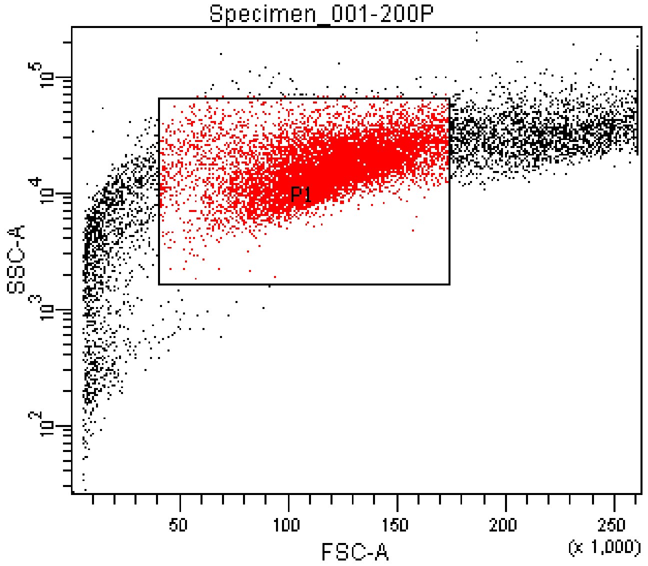

Experiment Name: 20180702 DHE TRI-3
Specimen Name: Specimen_001
Tube Name: 200P
Record Date: Jul 2, 2018 3:16:45 PM
$OP: Administrator
GUID: b115ceb8-6a27-487b-a1e7-5...

Population	#Events	%Parent	PI-A Mean
P1	10,000	68.2	148

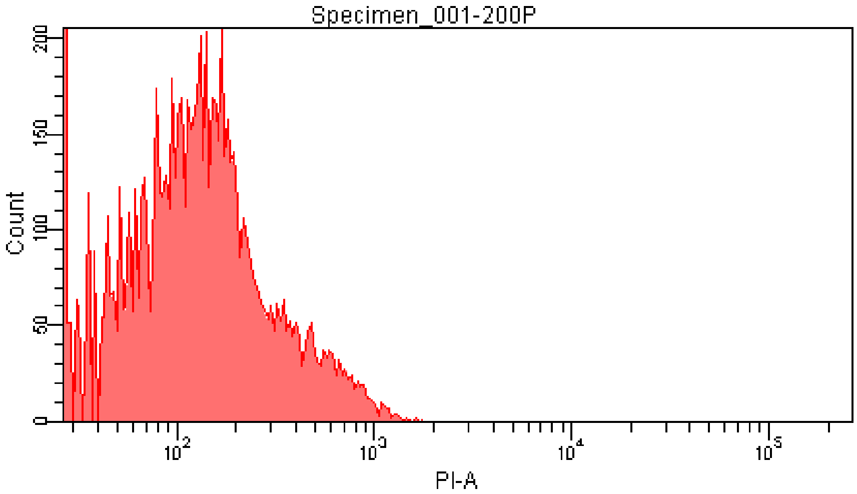

FACSDiva Version 6.1.3

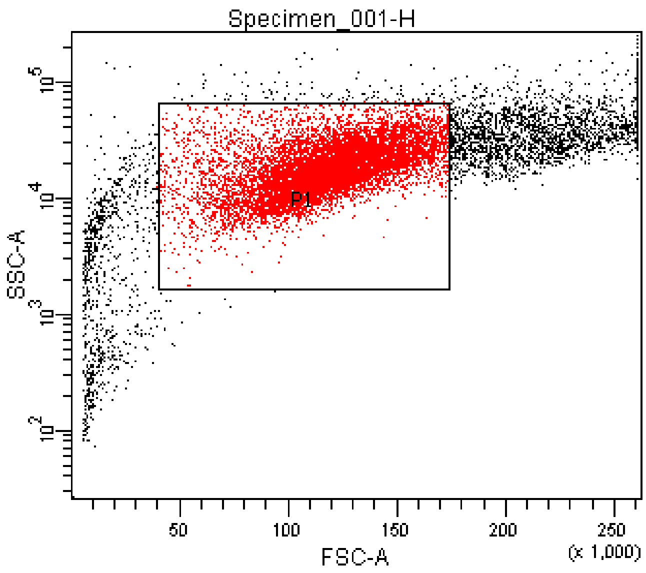

Experiment Name: 20180702 DHE TRI-3
Specimen Name: Specimen_001
Tube Name: H
Record Date: Jul 2, 2018 3:18:53 PM
$OP: Administrator
GUID: 38ed7e3e-5083-4cab-a694-2f...

Population	#Events	%Parent	PI-A Mean
P1	10,000	72.8	136

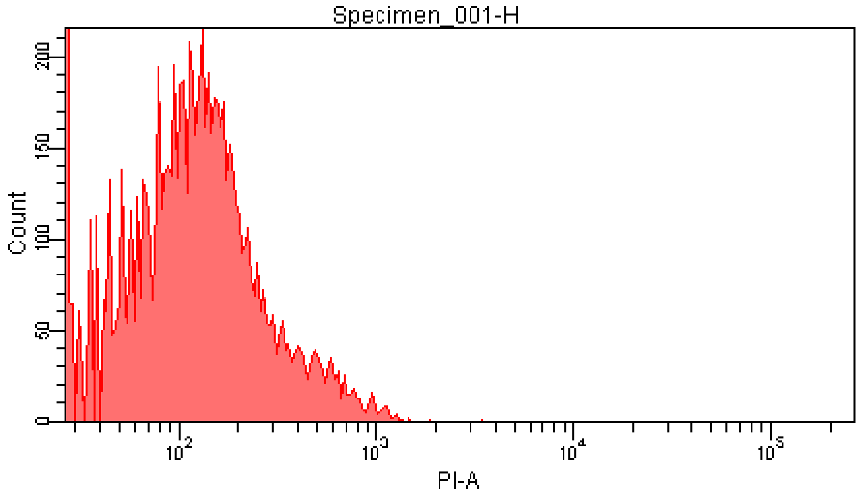

FACSDiva Version 6.1.3

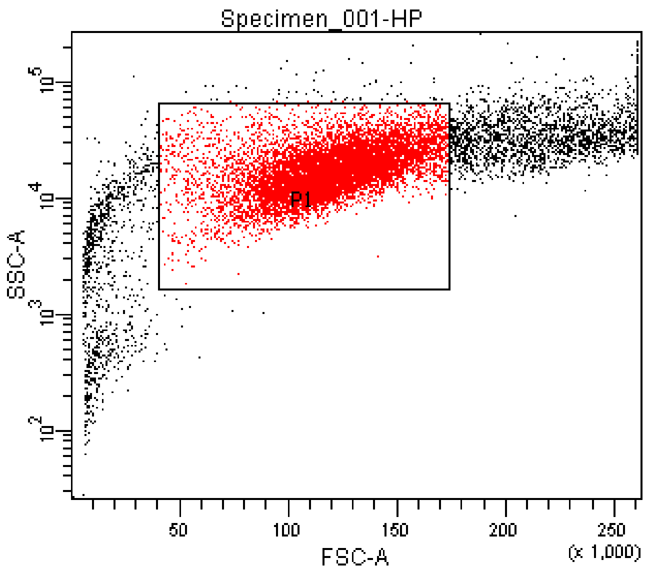

Experiment Name:	20180702 DHE TRI-3
Specimen Name:	Specimen_001
Tube Name:	HP
Record Date:	Jul 2, 2018 3:19:50 PM
$OP:	Administrator
GUID:	3665c5b1-d6d5-4dbf-ac6b-5f...

Population	#Events	%Parent	PI-A Mean
P1	10,000	72.1	137

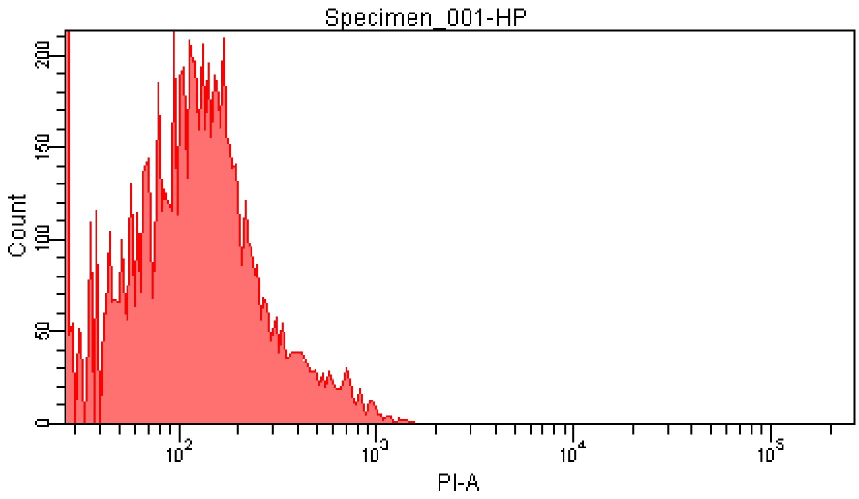

FACSDiva Version 6.1.3

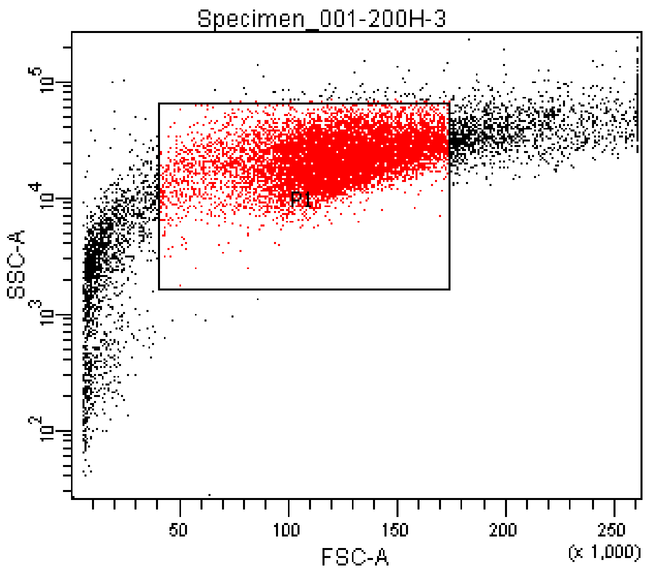

Experiment Name:	20180702 DHE TRI-3
Specimen Name:	Specimen_001
Tube Name:	200H-3
Record Date:	Jul 2, 2018 3:08:08 PM
$OP:	Administrator
GUID:	1f914075-2225-4b80-a845-4b...

Population	#Events	%Parent	PI-A Mean
P1	10,000	74.2	230

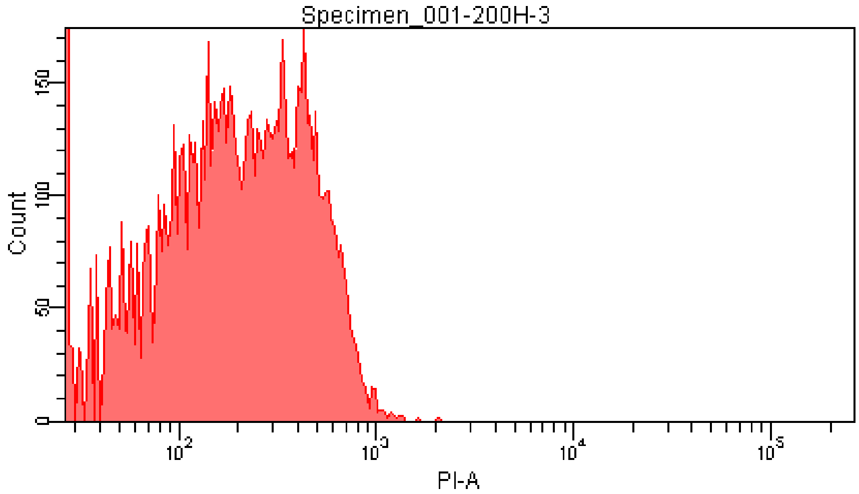

FACSDiva Version 6.1.3

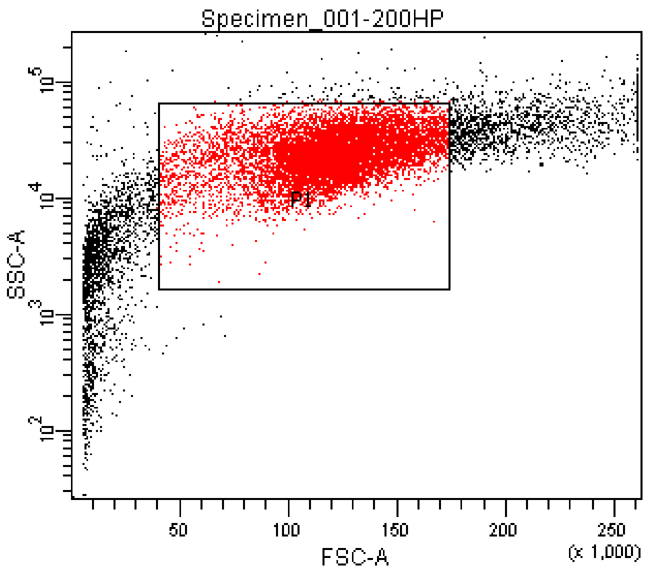

Experiment Name:	20180702 DHE TRI-3
Specimen Name:	Specimen_001
Tube Name:	200HP
Record Date:	Jul 2, 2018 3:15:32 PM
$OP:	Administrator
GUID:	de6a6bd1-1ece-4c49-a5f7-a1...

Population	#Events	%Parent	PI-A Mean
P1	10,000	72.9	291

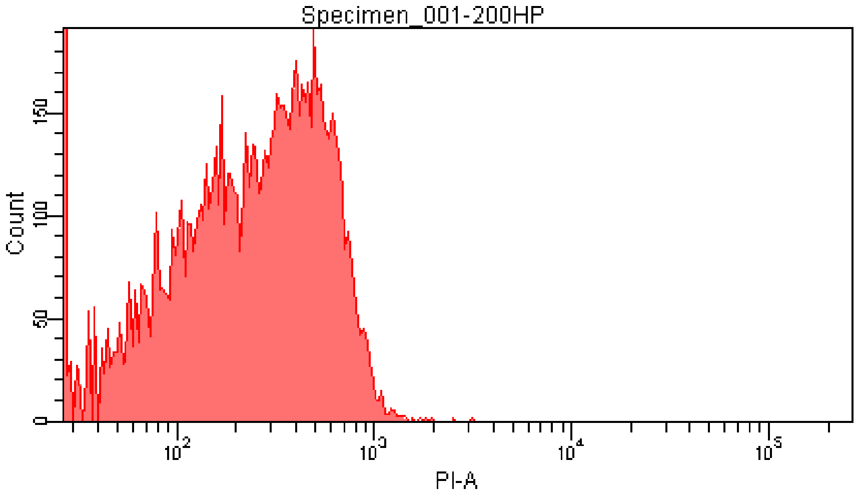

Supplement: S3 File — (PDF) [file pone.0222126.s005.pdf]
