# Supplementary material for: Application of non-invasive low-intensity pulsed electric field with thermal cycling-hyperthermia for synergistically enhanced anticancer effect of chlorogenic acid on PANC-1 cells
Source: PLoS One. 2020 Jan 29;15(1):e0222126. doi: 10.1371/journal.pone.0222126 (PMC6988950; doi:10.1371/journal.pone.0222126)
Supplement: Attachment — Submitted filename: Response to Reviewers.docx [file pone.0222126.s006.docx]

November 14th, 2019

Dr. Ferenc Gallyas, Jr.

*PLOS ONE*

1160 Battery Street

Koshland Building East, Suite 225

San Francisco, CA 94111, USA

Re: PONE-D-19-23209
 Application of non-invasive low-intensity pulsed electric field with thermal

cycling-hyperthermia for synergistically enhanced anticancer effect of chlorogenic

acid on PANC-1 cells

By: C. H. Lu, Y. Y. Kuo, K. B. Lin, W. T. Chen, and C. Y. Chao

Dear Editor Dr. Gallyas:

We are grateful to the reviewers for their comments and suggestions in this manuscript. In the revised manuscript, we have adopted the reviewers’ comments and suggestions, and believe the manuscript now is in a final form suitable for publication.

**Point-by-point responses to the reviewers’ comments on the manuscript**

**“Application of non-invasive low-intensity pulsed electric field with thermal cycling-hyperthermia for synergistically enhanced anticancer effect of chlorogenic acid on PANC-1 cells”**

**Reviewers’ comments to the Author**

**Reviewer #1**:
1)The paper proposes a novel treatment modality includes non-invasive treatment CGC, coupled with thermal-cycling hyperthermia (TC-HT) and low-intensity pulsedelectric field (LIPEF). The triple combination leads to excessive apoptotic cell death with known signatures for apoptosis in Panc-1 pancreatic cancer cells. Although MS includes non-malign cell data, due to genetic heterogenety of pancreatic tumors, other cell lines carry different mutations for p53 or KRas is missing. MS skips the mutational loss of functional p53 status of Panc-1 cells in discussion part. Therefore discussion part should be revised according to p53 burden in tumors and effect on therapy.

**Response:**

We thank the referee for this suggestion. We have added more information about p53 burden in tumors and effect on therapy in the “Discussion” part of the revised manuscript. Furthermore, another pancreatic cancer cell line AsPC-1 has been examined for the specificity of our treatment. The results show that AsPC-1 cells are resistant to p53-mediated cytotoxicity induced by the triple treatment (see **S1 Fig**). The data have also been included in the revised manuscript.

**Revised part:** Page 28_line 478-483.

Page 30_line 512-514.
 Page 37_line 577-678.

Page 41_line 793-795.

2)There are number of typo errors in the text.

**Response:**

We would like to thank the referee for this kind reminder. The typos have been corrected in the revised manuscript.
**Revised part:** Page 6_line 116.
 Page 14_line 245.

Page 19_line 334.
 Page 20_line 351.
 Page 21_line 366.

Page 22_line 387.

Page 26_line 454.

Page 30_line 524-526.

Page 32_line 547.

Page 33_line 578.

**Reviewer #2**:

Title: "non-invasive low-intensity pulsed electric field" - The description of PEF is lacking in the M&M and why use it in their studies. It is noteworthy that there are electric fields being larger fields (1.8 and 1.26 kV/cm) can produced a high drug intake rate during the first 10 ms that can decrease significantly thereafter. On the other hand, the intake rate during the first 10 ms for the two smaller fields (0.63 and 0.45 kV/cm) maybe undetectable and may gradually increased afterward. The rates for these two fields can be similar to one another until the end of the analysis. In addition, the optimal PEF levels for in vitro tests need to be described in detail; e.g., how many pulses at 1 kHz and 0.45 kV/cm to be nonlethal to normal cells but effectively kill cancer cells.
**Response:**

Thanks for the referee’s suggestion. The referee might have misunderstood our points in that we actually adopted electrical stimulation of capacitive coupling in our study rather than conductive coupling that uses electrodes in direct contact the biological sample under treatment [1]. Thus, LIPEF performed in this study is a non-invasively electrical stimulation by which the cells are exposed to electric field. The method of such non-invasive strategy of LIPEF is somewhat similar to that of tumor treating fields (TTFields) [2], but in a non-contact approach with different waveform and field strength. Moreover, there has been an *in vivo* study using contactless electrodes to investigate the biological effects of rat in response to the exposure of AC electric field at high voltages (15-20 kV) [3].

In our experimental setup, two copper parallel electrodes separated by an air gap present a non-contact means to build up an electric field passing through biological sample (see **Fig 1**). Our approach was mainly focused on the adoption of mild electric field strength to study the synergistic activity with CGA and TC-HT. For optimization of LIPEF parameters, we have studied different LIPEF field strength ranging from 30 to 90 V/cm. The new data have been incorporated in **Fig 2C** and **2D** to enhance the clarity of the revised manuscript. As shown in **Fig 2C**, we found that both 60 and 90 V/cm LIPEF were capable of increasing the anticancer cytotoxicity of the CGA combined with TC-HT in PANC-1 cells. Interestingly, the killing effect of the triple combination of CGA, TC-HT, and 60 V/cm LIPEF against PANC-1 cells was not observed in H6c7 pancreatic normal cells. However, the triple treatment with LIPEF at 90 V/cm was found to cause a significant decrease in the cell viability of H6c7 cells (see **Fig 2D**). Therefore, the field strength of LIPEF in combination with CGA and TC-HT against PANC-1 cells was chosen at 60 V/cm to conduct experiments in this study. Furthermore, the synergistic effect of triple treatment with different LIPEF frequency has also been examined. The obtained results have been shown in **S2 Fig**, which indicates a frequency-dependent reduction in cell viability of PANC-1 following the triple treatment, especially at 2 Hz. This investigation has been included in the “Results”, “Discussion”, and “Figure caption” parts of the revised manuscript.

**Revised part:** Page 8_line 138 ~ page 9_line 159.

Page 16_line 279-288.

Page 17_line 302-305.

Page 30_line 520-523.

Page 41_line 796-798.

Abstract: "In this study, we propose a novel non-invasive treatment calling for exposure of cells to CGA, coupled with thermal-cycling hyperthermia (TC-HT) and low-intensity pulsed electric field (LIPEF)." Why use non-invasive here? No definition for CGA here. The authors published a previous article (PLoS One. 2019; 14(5): e0217676.Published online 2019 May 31. doi: 10.1371/journal.pone.0217676) using CGA and TC-HT treatment of PANC1 cells. There is no rationale for using LIPEF in this study.

**Response:**

The reason for the term “non-invasive” used here is due to both thermal-cycling hyperthermia (TC-HT) and low-intensity pulsed electric field (LIPEF) are applied externally without direct contact with cells. Since it could be obscure for readers, we have rewritten this sentence and also added the definition of CGA in the abstract.

We have recently shown that the combination of CGA and TC-HT exhibited a synergistic cytotoxic effect in a moderate manner on PANC-1 cells. Based on the positive results of the application of LIPEF in triple remedy in our previous study, we attempt to employ non-contact LIPEF to combine with CGA and TC-HT as triple combination to improve the anticancer efficacy on PANC-1 cells. The rational for using LIPEF in this study has been added in the “Abstract” and “Introduction” parts of the revised manuscript.
**Revised part:** Page 2_line 35-40.

Page 4_line 74-81.

Introduction: As written, it does not provide a rationale for the study. The authors have published a paper on CGA and TC-HT which needs to be articulated at the beginning with the findings. Then, why use LIPEF as a combination treatment with CGA and TC-HT in this study. This section is poorly written. For example, "We have recently shown that the thermal cycling-hyperthermia (TC-HT) has an anticancer synergy via coupling with the polyphenols, chlorogenic acid (CGA) and epigallocatechin gallate (EGCG) in combating human pancreatic cancer PANC-1 cells [7], thanks to the effect of the cell cycle arrest and the induction of mitochondria-mediated apoptosis, which were not found in the separate treatments."

**Response:**

Thanks the referee for providing this comment. We have rephrased this section in the “Introduction” part of the revised manuscript to make the rationale of this study clear.
**Revised part:** Page 4_line 74-81.

Discussion: Poorly written. "Unlike alternating current signal stimulus, non-invasive LIPEF can be viewed as the composition of many sinusoidal subcomponents with multiple frequencies [30]. When the electric pluses enter and interact with a biological sample, we propose that the LIPEF is dispersed electrically to multiple frequencies due to the encounter with biological dielectrics. Thus, the LIPEF’s multiple frequencies, caused by electrical dispersion in biological dielectrics, can simultaneously interact with molecules, proteins, organelles, and cells to induce bioeffects during the LIPEF exposure. The study points out that the application of LIPEF could intensify the disturbance of mitochondria triggered by double treatment with CGA and TC-HT, which in turn stimulated the depolarization of MMP." Unfortunately, the description of LIPEF is lacking in the study which needs to be addressed in detail.

**Response:**

The sentences have been removed from this section and this paragraph in the revised manuscript has been modified as : “Previous studies have reported that the high-frequency components of PEF could interact with intermembrane organelles, such as mitochondria and nucleus, when PEF passes through cytomembrane [4, 5]. It indicates that the application of LIPEF could intensify the disturbance of mitochondria triggered by double treatment with CGA and TC-HT, which in turn stimulated the depolarization of MMP.”

The description and details of LIPEF have now been included in the “Materials and Methods” part of the revised manuscript.

**Revised part:** Page 8_line 138 ~ page 9_line 159.

Page 30_line 516-518.

**References:**

1.	Robinson VS, Garner AL, Loveless AM, Neculaes VB. Calculated plasma membrane voltage induced by applying electric pulses using capacitive coupling. Biomed Phys Eng Expr. 2017;3(2).

2.	Hottinger AF, Pacheco P, Stupp R. Tumor treating fields: a novel treatment modality and its use in brain tumors. Neuro-oncology. 2016;18(10):1338-49.

3.	Harakawa S, Inoue N, Hori T, Tochio K, Kariya T, Takahashi K, et al. Effects of a 50 Hz electric field on plasma lipid peroxide level and antioxidant activity in rats. Bioelectromagnetics. 2005;26(7):589-94.

4.	Kotnik T, Miklavcic D. Theoretical evaluation of voltage inducement on internal membranes of biological cells exposed to electric fields. Biophys J. 2006;90(2):480-91.

5.	Mi Y, Sun C, Yao C, Li C, Mo D, Tang L, et al. Effects of steep pulsed electric fields (SPEF) on mitochondrial transmembrane potential of human liver cancer cell. Conf Proc IEEE Eng Med Biol Soc. 2007;2007:5815-8.

Finally, we would like to thank the referees again for his/her constructive suggestions, which have been incorporated in the revised manuscript to improve the presentation. The revised manuscript has addressed the raised points by both referees, and we believe it should now be acceptable for publication.

Sincerely yours,

Chih-Yu Chao, Ph.D

Professor

Biomedical & Molecular Imaging Center

College of Medicine

National Taiwan University

Taipei 10617, Taiwan

Tel: +886-2-33665130; Fax: +886-2-33665088

E-mail: cychao@phys.ntu.edu.tw
