# Supplementary material for: Application of non-invasive low-intensity pulsed electric field with thermal cycling-hyperthermia for synergistically enhanced anticancer effect of chlorogenic acid on PANC-1 cells
Source: PLoS One. 2020 Jan 29;15(1):e0222126. doi: 10.1371/journal.pone.0222126 (PMC6988950; doi:10.1371/journal.pone.0222126)
Supplement: Attachment — Submitted filename: Response to Reviewers.docx [file pone.0222126.s007.docx]

December 7th, 2019

Dr. Ferenc Gallyas, Jr.

*PLOS ONE*

1160 Battery Street

Koshland Building East, Suite 225

San Francisco, CA 94111, USA

Re: PONE-D-19-23209R1
 Application of non-invasive low-intensity pulsed electric field with thermal

cycling-hyperthermia for synergistically enhanced anticancer effect of chlorogenic

acid on PANC-1 cells

By: C. H. Lu, Y. Y. Kuo, K. B. Lin, W. T. Chen, and C. Y. Chao

Dear Editor Dr. Gallyas:

We would like to thank you for the reminder. We have provided images for original blots that meet PLOS ONE's requirement, and believe the manuscript should now be acceptable for publication. The images are collected in a ZIP archive and uploaded as a supporting information file (**S1 File**) in the resubmission.

**Revised part:** Page 41_line 799.

Sincerely yours,

Chih-Yu Chao, Ph.D

Professor

Biomedical & Molecular Imaging Center

College of Medicine

National Taiwan University

Taipei 10617, Taiwan

Tel: +886-2-33665130; Fax: +886-2-33665088

E-mail: cychao@phys.ntu.edu.tw
